# Transforming jet flavour tagging at ATLAS

**DOI:** 10.1038/s41467-025-65059-6

**Published:** 2026-01-14

**Authors:** The ATLAS Collaboration, G. Aad, E. Aakvaag, B. Abbott, S. Abdelhameed, K. Abeling, N. J. Abicht, S. H. Abidi, M. Aboelela, A. Aboulhorma, H. Abramowicz, Y. Abulaiti, B. S. Acharya, A. Ackermann, C. Adam Bourdarios, L. Adamczyk, S. V. Addepalli, M. J. Addison, J. Adelman, A. Adiguzel, T. Adye, A. A. Affolder, Y. Afik, M. N. Agaras, A. Aggarwal, C. Agheorghiesei, F. Ahmadov, S. Ahuja, X. Ai, G. Aielli, A. Aikot, M. Ait Tamlihat, B. Aitbenchikh, M. Akbiyik, T. P. A. Åkesson, A. V. Akimov, D. Akiyama, N. N. Akolkar, S. Aktas, G. L. Alberghi, J. Albert, U. Alberti, P. Albicocco, G. L. Albouy, S. Alderweireldt, Z. L. Alegria, M. Aleksa, I. N. Aleksandrov, C. Alexa, T. Alexopoulos, F. Alfonsi, M. Algren, M. Alhroob, B. Ali, H. M. J. Ali, S. Ali, S. W. Alibocus, M. Aliev, G. Alimonti, W. Alkakhi, C. Allaire, B. M. M. Allbrooke, J. S. Allen, J. F. Allen, P. P. Allport, A. Aloisio, F. Alonso, C. Alpigiani, Z. M. K. Alsolami, A. Alvarez Fernandez, M. Alves Cardoso, M. G. Alviggi, M. Aly, Y. Amaral Coutinho, A. Ambler, C. Amelung, M. Amerl, C. G. Ames, T. Amezza, D. Amidei, B. Amini, K. Amirie, A. Amirkhanov, S. P. Amor Dos Santos, K. R. Amos, D. Amperiadou, S. An, C. Anastopoulos, T. Andeen, J. K. Anders, A. C. Anderson, A. Andreazza, S. Angelidakis, A. Angerami, A. V. Anisenkov, A. Annovi, C. Antel, E. Antipov, M. Antonelli, F. Anulli, M. Aoki, T. Aoki, M. A. Aparo, L. Aperio Bella, M. Apicella, C. Appelt, A. Apyan, M. Arampatzi, S. J. Arbiol Val, C. Arcangeletti, A. T. H. Arce, J-F. Arguin, S. Argyropoulos, J.-H. Arling, O. Arnaez, H. Arnold, G. Artoni, H. Asada, K. Asai, S. Asai, S. Asatryan, N. A. Asbah, R. A. Ashby Pickering, A. M. Aslam, K. Assamagan, R. Astalos, K. S. V. Astrand, S. Atashi, R. J. Atkin, H. Atmani, P. A. Atmasiddha, K. Augsten, A. D. Auriol, V. A. Austrup, G. Avolio, K. Axiotis, A. Azzam, D. Babal, H. Bachacou, K. Bachas, A. Bachiu, E. Bachmann, M. J. Backes, A. Badea, T. M. Baer, P. Bagnaia, M. Bahmani, D. Bahner, K. Bai, J. T. Baines, L. Baines, O. K. Baker, E. Bakos, D. Bakshi Gupta, L. E. Balabram Filho, V. Balakrishnan, R. Balasubramanian, E. M. Baldin, P. Balek, E. Ballabene, F. Balli, L. M. Baltes, W. K. Balunas, J. Balz, I. Bamwidhi, E. Banas, M. Bandieramonte, A. Bandyopadhyay, S. Bansal, L. Barak, M. Barakat, E. L. Barberio, D. Barberis, M. Barbero, M. Z. Barel, T. Barillari, M-S. Barisits, T. Barklow, P. Baron, D. A. Baron Moreno, A. Baroncelli, A. J. Barr, J. D. Barr, F. Barreiro, J. Barreiro Guimarães da Costa, M. G. Barros Teixeira, S. Barsov, F. Bartels, R. Bartoldus, A. E. Barton, P. Bartos, A. Basan, M. Baselga, S. Bashiri, A. Bassalat, M. J. Basso, S. Bataju, R. Bate, R. L. Bates, S. Batlamous, M. Battaglia, D. Battulga, M. Bauce, M. Bauer, P. Bauer, L. T. Bayer, L. T. Bazzano Hurrell, J. B. Beacham, T. Beau, J. Y. Beaucamp, P. H. Beauchemin, P. Bechtle, H. P. Beck, K. Becker, A. J. Beddall, V. A. Bednyakov, C. P. Bee, L. J. Beemster, M. Begalli, M. Begel, J. K. Behr, J. F. Beirer, F. Beisiegel, M. Belfkir, G. Bella, L. Bellagamba, A. Bellerive, C. D. Bellgraph, P. Bellos, K. Beloborodov, D. Benchekroun, F. Bendebba, Y. Benhammou, K. C. Benkendorfer, L. Beresford, M. Beretta, E. Bergeaas Kuutmann, N. Berger, B. Bergmann, J. Beringer, G. Bernardi, C. Bernius, F. U. Bernlochner, F. Bernon, A. Berrocal Guardia, T. Berry, P. Berta, A. Berthold, A. Berti, R. Bertrand, S. Bethke, A. Betti, A. J. Bevan, L. Bezio, N. K. Bhalla, S. Bharthuar, S. Bhatta, P. Bhattarai, Z. M. Bhatti, K. D. Bhide, V. S. Bhopatkar, R. M. Bianchi, G. Bianco, O. Biebel, M. Biglietti, C. S. Billingsley, Y. Bimgdi, M. Bindi, A. Bingham, A. Bingul, C. Bini, G. A. Bird, M. Birman, M. Biros, S. Biryukov, T. Bisanz, E. Bisceglie, J. P. Biswal, D. Biswas, I. Bloch, A. Blue, U. Blumenschein, J. Blumenthal, V. S. Bobrovnikov, L. Boccardo, M. Boehler, B. Boehm, D. Bogavac, A. G. Bogdanchikov, L. S. Boggia, V. Boisvert, P. Bokan, T. Bold, M. Bomben, M. Bona, M. Boonekamp, A. G. Borbély, I. S. Bordulev, G. Borissov, D. Bortoletto, D. Boscherini, M. Bosman, K. Bouaouda, N. Bouchhar, L. Boudet, J. Boudreau, E. V. Bouhova-Thacker, D. Boumediene, R. Bouquet, A. Boveia, J. Boyd, D. Boye, I. R. Boyko, L. Bozianu, J. Bracinik, N. Brahimi, G. Brandt, O. Brandt, B. Brau, J. E. Brau, R. Brener, L. Brenner, R. Brenner, S. Bressler, G. Brianti, D. Britton, D. Britzger, I. Brock, R. Brock, G. Brooijmans, A. J. Brooks, E. M. Brooks, E. Brost, L. M. Brown, L. E. Bruce, T. L. Bruckler, P. A. Bruckman de Renstrom, B. Brüers, A. Bruni, G. Bruni, D. Brunner, M. Bruschi, N. Bruscino, T. Buanes, Q. Buat, D. Buchin, A. G. Buckley, O. Bulekov, B. A. Bullard, S. Burdin, C. D. Burgard, A. M. Burger, B. Burghgrave, O. Burlayenko, J. Burleson, J. C. Burzynski, E. L. Busch, V. Büscher, P. J. Bussey, J. M. Butler, C. M. Buttar, J. M. Butterworth, W. Buttinger, C. J. Buxo Vazquez, A. R. Buzykaev, S. Cabrera Urbán, L. Cadamuro, D. Caforio, H. Cai, Y. Cai, Y. Cai, V. M. M. Cairo, O. Cakir, N. Calace, P. Calafiura, G. Calderini, P. Calfayan, L. Calic, G. Callea, L. P. Caloba, D. Calvet, S. Calvet, R. Camacho Toro, S. Camarda, D. Camarero Munoz, P. Camarri, C. Camincher, M. Campanelli, A. Camplani, V. Canale, A. C. Canbay, E. Canonero, J. Cantero, Y. Cao, F. Capocasa, M. Capua, A. Carbone, R. Cardarelli, J. C. J. Cardenas, M. P. Cardiff, G. Carducci, T. Carli, G. Carlino, J. I. Carlotto, B. T. Carlson, E. M. Carlson, J. Carmignani, L. Carminati, A. Carnelli, M. Carnesale, S. Caron, E. Carquin, I. B. Carr, S. Carrá, G. Carratta, A. M. Carroll, M. P. Casado, P. Casolaro, M. Caspar, F. L. Castillo, L. Castillo Garcia, V. Castillo Gimenez, N. F. Castro, A. Catinaccio, J. R. Catmore, T. Cavaliere, V. Cavaliere, L. J. Caviedes Betancourt, E. Celebi, S. Cella, V. Cepaitis, K. Cerny, A. S. Cerqueira, A. Cerri, L. Cerrito, F. Cerutti, B. Cervato, A. Cervelli, G. Cesarini, S. A. Cetin, P. M. Chabrillat, R. Chakkappai, S. Chakraborty, J. Chan, W. Y. Chan, J. D. Chapman, E. Chapon, B. Chargeishvili, D. G. Charlton, C. Chauhan, Y. Che, S. Chekanov, S. V. Chekulaev, G. A. Chelkov, B. Chen, B. Chen, H. Chen, H. Chen, J. Chen, J. Chen, M. Chen, S. Chen, S. J. Chen, X. Chen, X. Chen, Z. Chen, C. L. Cheng, H. C. Cheng, S. Cheong, A. Cheplakov, E. Cherepanova, R. Cherkaoui El Moursli, E. Cheu, K. Cheung, L. Chevalier, V. Chiarella, G. Chiarelli, G. Chiodini, A. S. Chisholm, A. Chitan, M. Chitishvili, M. V. Chizhov, K. Choi, Y. Chou, E. Y. S. Chow, K. L. Chu, M. C. Chu, X. Chu, Z. Chubinidze, J. Chudoba, J. J. Chwastowski, D. Cieri, K. M. Ciesla, V. Cindro, A. Ciocio, F. Cirotto, Z. H. Citron, M. Citterio, D. A. Ciubotaru, A. Clark, P. J. Clark, N. Clarke Hall, C. Clarry, S. E. Clawson, C. Clement, Y. Coadou, M. Cobal, A. Coccaro, R. F. Coelho Barrue, R. Coelho Lopes De Sa, S. Coelli, L. S. Colangeli, B. Cole, P. Collado Soto, J. Collot, R. Coluccia, P. Conde Muiño, M. P. Connell, S. H. Connell, E. I. Conroy, M. Contreras Cossio, F. Conventi, A. M. Cooper-Sarkar, L. Corazzina, F. A. Corchia, A. Cordeiro Oudot Choi, L. D. Corpe, M. Corradi, F. Corriveau, A. Cortes-Gonzalez, M. J. Costa, F. Costanza, D. Costanzo, B. M. Cote, J. Couthures, G. Cowan, K. Cranmer, L. Cremer, D. Cremonini, S. Crépé-Renaudin, F. Crescioli, T. Cresta, M. Cristinziani, M. Cristoforetti, V. Croft, J. E. Crosby, G. Crosetti, A. Cueto, H. Cui, Z. Cui, B. M. Cunnett, W. R. Cunningham, F. Curcio, J. R. Curran, M. J. Da Cunha Sargedas De Sousa, J. V. Da Fonseca Pinto, C. Da Via, W. Dabrowski, T. Dado, S. Dahbi, T. Dai, D. Dal Santo, C. Dallapiccola, M. Dam, G. D’amen, V. D’Amico, J. Damp, J. R. Dandoy, M. D’Andrea, D. Dannheim, G. D’anniballe, M. Danninger, V. Dao, G. Darbo, S. J. Das, F. Dattola, S. D’Auria, A. D’Avanzo, T. Davidek, J. Davidson, I. Dawson, K. De, C. De Almeida Rossi, R. De Asmundis, N. De Biase, S. De Castro, N. De Groot, P. de Jong, H. De la Torre, A. De Maria, A. De Salvo, U. De Sanctis, F. De Santis, A. De Santo, J. B. De Vivie De Regie, J. Debevc, D. V. Dedovich, J. Degens, A. M. Deiana, J. Del Peso, L. Delagrange, F. Deliot, C. M. Delitzsch, M. Della Pietra, D. Della Volpe, A. Dell’Acqua, L. Dell’Asta, M. Delmastro, C. C. Delogu, P. A. Delsart, S. Demers, M. Demichev, S. P. Denisov, H. Denizli, L. D’Eramo, D. Derendarz, F. Derue, P. Dervan, A. M. Desai, K. Desch, F. A. Di Bello, A. Di Ciaccio, L. Di Ciaccio, A. Di Domenico, C. Di Donato, A. Di Girolamo, G. Di Gregorio, A. Di Luca, B. Di Micco, R. Di Nardo, K. F. Di Petrillo, M. Diamantopoulou, F. A. Dias, M. A. Diaz, A. R. Didenko, M. Didenko, S. D. Diefenbacher, E. B. Diehl, S. Díez Cornell, C. Diez Pardos, C. Dimitriadi, A. Dimitrievska, A. Dimri, Y. Ding, J. Dingfelder, T. Dingley, I-M. Dinu, S. J. Dittmeier, F. Dittus, M. Divisek, B. Dixit, F. Djama, T. Djobava, C. Doglioni, A. Dohnalova, Z. Dolezal, K. Domijan, K. M. Dona, M. Donadelli, B. Dong, J. Donini, A. D’Onofrio, M. D’Onofrio, J. Dopke, A. Doria, N. Dos Santos Fernandes, I. A. Dos Santos Luz, P. Dougan, M. T. Dova, A. T. Doyle, M. A. Draguet, M. P. Drescher, E. Dreyer, I. Drivas-koulouris, M. Drnevich, M. Drozdova, D. Du, T. A. du Pree, Z. Duan, M. Dubau, F. Dubinin, M. Dubovsky, E. Duchovni, G. Duckeck, P. K. Duckett, O. A. Ducu, D. Duda, A. Dudarev, E. R. Duden, M. D’uffizi, L. Duflot, M. Dührssen, I. Duminica, A. E. Dumitriu, M. Dunford, S. Dungs, K. Dunne, A. Duperrin, H. Duran Yildiz, M. Düren, A. Durglishvili, D. Duvnjak, G. I. Dyckes, M. Dyndal, B. S. Dziedzic, Z. O. Earnshaw, G. H. Eberwein, B. Eckerova, S. Eggebrecht, E. Egidio Purcino De Souza, G. Eigen, K. Einsweiler, T. Ekelof, P. A. Ekman, S. El Farkh, Y. El Ghazali, H. El Jarrari, A. El Moussaouy, V. Ellajosyula, M. Ellert, F. Ellinghaus, T. A. Elliot, N. Ellis, J. Elmsheuser, M. Elsawy, M. Elsing, D. Emeliyanov, Y. Enari, I. Ene, S. Epari, D. Ernani Martins Neto, F. Ernst, M. Errenst, M. Escalier, C. Escobar, E. Etzion, G. Evans, H. Evans, L. S. Evans, A. Ezhilov, S. Ezzarqtouni, F. Fabbri, L. Fabbri, G. Facini, V. Fadeyev, R. M. Fakhrutdinov, D. Fakoudis, S. Falciano, L. F. Falda Ulhoa Coelho, F. Fallavollita, G. Falsetti, J. Faltova, C. Fan, K. Y. Fan, Y. Fan, Y. Fang, M. Fanti, M. Faraj, Z. Farazpay, A. Farbin, A. Farilla, K. Farman, T. Farooque, J. N. Farr, S. M. Farrington, F. Fassi, D. Fassouliotis, L. Fayard, P. Federic, P. Federicova, O. L. Fedin, M. Feickert, L. Feligioni, D. E. Fellers, C. Feng, Z. Feng, M. J. Fenton, L. Ferencz, B. Fernandez Barbadillo, P. Fernandez Martinez, M. J. V. Fernoux, J. Ferrando, A. Ferrari, P. Ferrari, R. Ferrari, D. Ferrere, C. Ferretti, M. P. Fewell, D. Fiacco, F. Fiedler, P. Fiedler, S. Filimonov, M. S. Filip, A. Filipčič, E. K. Filmer, F. Filthaut, M. C. N. Fiolhais, L. Fiorini, W. C. Fisher, T. Fitschen, P. M. Fitzhugh, I. Fleck, P. Fleischmann, T. Flick, M. Flores, L. R. Flores Castillo, L. Flores Sanz De Acedo, F. M. Follega, N. Fomin, J. H. Foo, A. Formica, A. C. Forti, E. Fortin, A. W. Fortman, L. Foster, L. Fountas, D. Fournier, H. Fox, P. Francavilla, S. Francescato, S. Franchellucci, M. Franchini, S. Franchino, D. Francis, L. Franco, V. Franco Lima, L. Franconi, M. Franklin, G. Frattari, Y. Y. Frid, J. Friend, N. Fritzsche, A. Froch, D. Froidevaux, J. A. Frost, Y. Fu, S. Fuenzalida Garrido, M. Fujimoto, K. Y. Fung, E. Furtado De Simas Filho, M. Furukawa, J. Fuster, A. Gaa, A. Gabrielli, A. Gabrielli, P. Gadow, G. Gagliardi, L. G. Gagnon, S. Gaid, S. Galantzan, J. Gallagher, E. J. Gallas, A. L. Gallen, B. J. Gallop, K. K. Gan, S. Ganguly, Y. Gao, A. Garabaglu, F. M. Garay Walls, C. García, A. Garcia Alonso, A. G. Garcia Caffaro, J. E. García Navarro, M. A. Garcia Ruiz, M. Garcia-Sciveres, G. L. Gardner, R. W. Gardner, N. Garelli, R. B. Garg, J. M. Gargan, C. A. Garner, C. M. Garvey, V. K. Gassmann, G. Gaudio, V. Gautam, P. Gauzzi, J. Gavranovic, I. L. Gavrilenko, A. Gavrilyuk, C. Gay, G. Gaycken, E. N. Gazis, A. Gekow, C. Gemme, M. H. Genest, A. D. Gentry, S. George, T. Geralis, A. A. Gerwin, P. Gessinger-Befurt, M. E. Geyik, M. Ghani, K. Ghorbanian, A. Ghosal, A. Ghosh, A. Ghosh, B. Giacobbe, S. Giagu, T. Giani, A. Giannini, S. M. Gibson, M. Gignac, D. T. Gil, A. K. Gilbert, B. J. Gilbert, D. Gillberg, G. Gilles, D. M. Gingrich, M. P. Giordani, P. F. Giraud, G. Giugliarelli, D. Giugni, F. Giuli, I. Gkialas, L. K. Gladilin, C. Glasman, M. Glazewska, R. M. Gleason, G. Glemža, M. Glisic, I. Gnesi, Y. Go, M. Goblirsch-Kolb, B. Gocke, D. Godin, B. Gokturk, S. Goldfarb, T. Golling, M. G. D. Gololo, D. Golubkov, J. P. Gombas, A. Gomes, G. Gomes Da Silva, A. J. Gomez Delegido, R. Gonçalo, L. Gonella, A. Gongadze, F. Gonnella, J. L. Gonski, R. Y. González Andana, S. González de la Hoz, M. V. Gonzalez Rodrigues, R. Gonzalez Suarez, S. Gonzalez-Sevilla, L. Goossens, B. Gorini, E. Gorini, A. Gorišek, T. C. Gosart, A. T. Goshaw, M. I. Gostkin, S. Goswami, C. A. Gottardo, S. A. Gotz, M. Gouighri, A. G. Goussiou, N. Govender, R. P. Grabarczyk, I. Grabowska-Bold, K. Graham, E. Gramstad, S. Grancagnolo, C. M. Grant, P. M. Gravila, F. G. Gravili, H. M. Gray, M. Greco, M. J. Green, C. Grefe, A. S. Grefsrud, I. M. Gregor, K. T. Greif, P. Grenier, S. G. Grewe, A. A. Grillo, K. Grimm, S. Grinstein, J.-F. Grivaz, E. Gross, J. Grosse-Knetter, L. Guan, G. Guerrieri, D. Guest, R. Guevara, R. Gugel, J. A. M. Guhit, A. Guida, E. Guilloton, S. Guindon, F. Guo, J. Guo, L. Guo, L. Guo, Y. Guo, A. Gupta, R. Gupta, S. Gupta, S. Gurbuz, S. S. Gurdasani, G. Gustavino, P. Gutierrez, L. F. Gutierrez Zagazeta, M. Gutsche, C. Gutschow, C. Gwenlan, C. B. Gwilliam, E. S. Haaland, A. Haas, M. Habedank, C. Haber, H. K. Hadavand, A. Haddad, A. Hadef, A. I. Hagan, J. J. Hahn, E. H. Haines, M. Haleem, J. Haley, G. D. Hallewell, L. Halser, K. Hamano, H. Hamdaoui, M. Hamer, S. E. D. Hammoud, E. J. Hampshire, J. Han, L. Han, L. Han, S. Han, K. Hanagaki, M. Hance, D. A. Hangal, H. Hanif, M. D. Hank, J. B. Hansen, P. H. Hansen, D. Harada, T. Harenberg, S. Harkusha, M. L. Harris, Y. T. Harris, J. Harrison, N. M. Harrison, P. F. Harrison, M. L. E. Hart, N. M. Hartman, N. M. Hartmann, R. Z. Hasan, Y. Hasegawa, F. Haslbeck, S. Hassan, R. Hauser, M. Haviernik, C. M. Hawkes, R. J. Hawkings, Y. Hayashi, D. Hayden, C. Hayes, R. L. Hayes, C. P. Hays, J. M. Hays, H. S. Hayward, M. He, Y. He, Y. He, N. B. Heatley, V. Hedberg, C. Heidegger, K. K. Heidegger, J. Heilman, S. Heim, T. Heim, J. G. Heinlein, J. J. Heinrich, L. Heinrich, J. Hejbal, M. Helbig, A. Held, S. Hellesund, C. M. Helling, S. Hellman, A. M. Henriques Correia, H. Herde, Y. Hernández Jiménez, L. M. Herrmann, T. Herrmann, G. Herten, R. Hertenberger, L. Hervas, M. E. Hesping, N. P. Hessey, J. Hessler, M. Hidaoui, N. Hidic, E. Hill, T. S. Hillersoy, S. J. Hillier, J. R. Hinds, F. Hinterkeuser, M. Hirose, S. Hirose, D. Hirschbuehl, T. G. Hitchings, B. Hiti, J. Hobbs, R. Hobincu, N. Hod, A. M. Hodges, M. C. Hodgkinson, B. H. Hodkinson, A. Hoecker, D. D. Hofer, J. Hofer, M. Holzbock, L. B. A. H. Hommels, V. Homsak, B. P. Honan, J. J. Hong, T. M. Hong, B. H. Hooberman, W. H. Hopkins, M. C. Hoppesch, Y. Horii, M. E. Horstmann, S. Hou, M. R. Housenga, A. S. Howard, J. Howarth, J. Hoya, M. Hrabovsky, T. Hryn’ova, P. J. Hsu, S.-C. Hsu, T. Hsu, M. Hu, Q. Hu, S. Huang, X. Huang, Y. Huang, Y. Huang, Y. Huang, Y. Huang, Z. Huang, Z. Hubacek, M. Huebner, F. Huegging, T. B. Huffman, M. Hufnagel Maranha De Faria, C. A. Hugli, M. Huhtinen, S. K. Huiberts, R. Hulsken, C. E. Hultquist, D. L. Humphreys, N. Huseynov, J. Huston, J. Huth, R. Hyneman, G. Iacobucci, G. Iakovidis, L. Iconomidou-Fayard, J. P. Iddon, P. Iengo, R. Iguchi, Y. Iiyama, T. Iizawa, Y. Ikegami, D. Iliadis, N. Ilic, H. Imam, G. Inacio Goncalves, S. A. Infante Cabanas, T. Ingebretsen Carlson, J. M. Inglis, G. Introzzi, M. Iodice, V. Ippolito, R. K. Irwin, M. Ishino, W. Islam, C. Issever, S. Istin, K. Itabashi, H. Ito, R. Iuppa, A. Ivina, V. Izzo, P. Jacka, P. Jackson, P. Jain, K. Jakobs, T. Jakoubek, J. Jamieson, W. Jang, S. Jankovych, M. Javurkova, P. Jawahar, L. Jeanty, J. Jejelava, P. Jenni, C. E. Jessiman, C. Jia, H. Jia, J. Jia, X. Jia, Z. Jia, C. Jiang, Q. Jiang, S. Jiggins, M. Jimenez Ortega, J. Jimenez Pena, S. Jin, A. Jinaru, O. Jinnouchi, P. Johansson, K. A. Johns, J. W. Johnson, F. A. Jolly, D. M. Jones, E. Jones, K. S. Jones, P. Jones, R. W. L. Jones, T. J. Jones, H. L. Joos, R. Joshi, J. Jovicevic, X. Ju, J. J. Junggeburth, T. Junkermann, A. Juste Rozas, M. K. Juzek, S. Kabana, A. Kaczmarska, M. Kado, H. Kagan, M. Kagan, A. Kahn, C. Kahra, T. Kaji, E. Kajomovitz, N. Kakati, N. Kakoty, I. Kalaitzidou, S. Kandel, N. J. Kang, D. Kar, K. Karava, E. Karentzos, O. Karkout, S. N. Karpov, Z. M. Karpova, V. Kartvelishvili, A. N. Karyukhin, E. Kasimi, J. Katzy, S. Kaur, K. Kawade, M. P. Kawale, C. Kawamoto, T. Kawamoto, E. F. Kay, F. I. Kaya, S. Kazakos, V. F. Kazanin, J. M. Keaveney, R. Keeler, G. V. Kehris, J. S. Keller, J. M. Kelly, J. J. Kempster, O. Kepka, J. Kerr, B. P. Kerridge, B. P. Kerševan, L. Keszeghova, R. A. Khan, A. Khanov, A. G. Kharlamov, T. Kharlamova, E. E. Khoda, M. Kholodenko, T. J. Khoo, G. Khoriauli, Y. Khoulaki, J. Khubua, Y. A. R. Khwaira, B. Kibirige, D. Kim, D. W. Kim, Y. K. Kim, N. Kimura, M. K. Kingston, A. Kirchhoff, C. Kirfel, F. Kirfel, J. Kirk, A. E. Kiryunin, S. Kita, O. Kivernyk, M. Klassen, C. Klein, L. Klein, M. H. Klein, S. B. Klein, U. Klein, A. Klimentov, T. Klioutchnikova, P. Kluit, S. Kluth, E. Kneringer, T. M. Knight, A. Knue, M. Kobel, D. Kobylianskii, S. F. Koch, M. Kocian, P. Kodyš, D. M. Koeck, T. Koffas, O. Kolay, I. Koletsou, T. Komarek, K. Köneke, A. X. Y. Kong, T. Kono, N. Konstantinidis, P. Kontaxakis, B. Konya, R. Kopeliansky, S. Koperny, K. Korcyl, K. Kordas, A. Korn, S. Korn, I. Korolkov, N. Korotkova, B. Kortman, O. Kortner, S. Kortner, W. H. Kostecka, M. Kostov, V. V. Kostyukhin, A. Kotsokechagia, A. Kotwal, A. Koulouris, A. Kourkoumeli-Charalampidi, C. Kourkoumelis, E. Kourlitis, O. Kovanda, R. Kowalewski, W. Kozanecki, A. S. Kozhin, V. A. Kramarenko, G. Kramberger, P. Kramer, M. W. Krasny, A. Krasznahorkay, A. C. Kraus, J. W. Kraus, J. A. Kremer, N. B. Krengel, T. Kresse, L. Kretschmann, J. Kretzschmar, P. Krieger, K. Krizka, K. Kroeninger, H. Kroha, J. Kroll, J. Kroll, K. S. Krowpman, U. Kruchonak, H. Krüger, N. Krumnack, M. C. Kruse, O. Kuchinskaia, S. Kuday, S. Kuehn, R. Kuesters, T. Kuhl, V. Kukhtin, Y. Kulchitsky, S. Kuleshov, J. Kull, E. V. Kumar, M. Kumar, N. Kumari, P. Kumari, A. Kupco, T. Kupfer, A. Kupich, O. Kuprash, H. Kurashige, L. L. Kurchaninov, O. Kurdysh, Y. A. Kurochkin, A. Kurova, M. Kuze, A. K. Kvam, J. Kvita, N. G. Kyriacou, C. Lacasta, F. Lacava, H. Lacker, D. Lacour, N. N. Lad, E. Ladygin, A. Lafarge, B. Laforge, T. Lagouri, F. Z. Lahbabi, S. Lai, W. S. Lai, J. E. Lambert, S. Lammers, W. Lampl, C. Lampoudis, G. Lamprinoudis, A. N. Lancaster, E. Lançon, U. Landgraf, M. P. J. Landon, V. S. Lang, O. K. B. Langrekken, A. J. Lankford, F. Lanni, K. Lantzsch, A. Lanza, M. Lanzac Berrocal, J. F. Laporte, T. Lari, D. Larsen, L. Larson, F. Lasagni Manghi, M. Lassnig, S. D. Lawlor, R. Lazaridou, M. Lazzaroni, H. D. M. Le, E. M. Le Boulicaut, L. T. Le Pottier, B. Leban, F. Ledroit-Guillon, T. F. Lee, L. L. Leeuw, M. Lefebvre, C. Leggett, G. Lehmann Miotto, M. Leigh, W. A. Leight, W. Leinonen, A. Leisos, M. A. L. Leite, C. E. Leitgeb, R. Leitner, K. J. C. Leney, T. Lenz, S. Leone, C. Leonidopoulos, A. Leopold, J. H. Lepage Bourbonnais, R. Les, C. G. Lester, M. Levchenko, J. Levêque, L. J. Levinson, G. Levrini, M. P. Lewicki, C. Lewis, D. J. Lewis, L. Lewitt, A. Li, B. Li, C. Li, C-Q. Li, H. Li, H. Li, H. Li, H. Li, J. Li, K. Li, L. Li, R. Li, S. Li, S. Li, T. Li, X. Li, Z. Li, Z. Li, Z. Li, S. Liang, Z. Liang, M. Liberatore, B. Liberti, K. Lie, J. Lieber Marin, H. Lien, H. Lin, S. F. Lin, L. Linden, R. E. Lindley, J. H. Lindon, J. Ling, E. Lipeles, A. Lipniacka, A. Lister, J. D. Little, B. Liu, B. X. Liu, D. Liu, D. Liu, E. H. L. Liu, J. K. K. Liu, K. Liu, K. Liu, M. Liu, M. Y. Liu, P. Liu, Q. Liu, X. Liu, X. Liu, Y. Liu, Y. L. Liu, Y. W. Liu, Z. Liu, S. L. Lloyd, E. M. Lobodzinska, P. Loch, E. Lodhi, T. Lohse, K. Lohwasser, E. Loiacono, J. D. Lomas, J. D. Long, I. Longarini, R. Longo, A. Lopez Solis, N. A. Lopez-canelas, N. Lorenzo Martinez, A. M. Lory, M. Losada, G. Löschcke Centeno, X. Lou, X. Lou, A. Lounis, P. A. Love, M. Lu, S. Lu, Y. J. Lu, H. J. Lubatti, C. Luci, F. L. Lucio Alves, F. Luehring, B. S. Lunday, O. Lundberg, J. Lunde, N. A. Luongo, M. S. Lutz, A. B. Lux, D. Lynn, R. Lysak, V. Lysenko, E. Lytken, V. Lyubushkin, T. Lyubushkina, M. M. Lyukova, M. Firdaus M. Soberi, H. Ma, K. Ma, L. L. Ma, W. Ma, Y. Ma, J. C. MacDonald, P. C. Machado De Abreu Farias, R. Madar, T. Madula, J. Maeda, T. Maeno, P. T. Mafa, H. Maguire, M. Maheshwari, V. Maiboroda, A. Maio, K. Maj, O. Majersky, S. Majewski, R. Makhmanazarov, N. Makovec, V. Maksimovic, B. Malaescu, J. Malamant, Pa. Malecki, V. P. Maleev, F. Malek, M. Mali, D. Malito, U. Mallik, A. Maloizel, S. Maltezos, A. Malvezzi Lopes, S. Malyukov, J. Mamuzic, G. Mancini, M. N. Mancini, G. Manco, J. P. Mandalia, S. S. Mandarry, I. Mandić, L. Manhaes de Andrade Filho, I. M. Maniatis, J. Manjarres Ramos, D. C. Mankad, A. Mann, T. Manoussos, M. N. Mantinan, S. Manzoni, L. Mao, X. Mapekula, A. Marantis, R. R. Marcelo Gregorio, G. Marchiori, M. Marcisovsky, C. Marcon, E. Maricic, M. Marinescu, S. Marium, M. Marjanovic, A. Markhoos, M. Markovitch, M. K. Maroun, G. T. Marsden, E. J. Marshall, Z. Marshall, S. Marti-Garcia, J. Martin, T. A. Martin, V. J. Martin, B. Martin dit Latour, L. Martinelli, M. Martinez, P. Martinez Agullo, V. I. Martinez Outschoorn, P. Martinez Suarez, S. Martin-Haugh, G. Martinovicova, V. S. Martoiu, A. C. Martyniuk, A. Marzin, D. Mascione, L. Masetti, J. Masik, A. L. Maslennikov, S. L. Mason, P. Massarotti, P. Mastrandrea, A. Mastroberardino, T. Masubuchi, T. T. Mathew, J. Matousek, D. M. Mattern, J. Maurer, T. Maurin, A. J. Maury, B. Maček, C. Mavungu Tsava, D. A. Maximov, A. E. May, E. Mayer, R. Mazini, I. Maznas, S. M. Mazza, E. Mazzeo, J. P. Mc Gowan, S. P. Mc Kee, C. A. Mc Lean, C. C. McCracken, E. F. McDonald, A. E. McDougall, L. F. Mcelhinney, J. A. Mcfayden, R. P. McGovern, R. P. Mckenzie, T. C. Mclachlan, D. J. Mclaughlin, S. J. McMahon, C. M. Mcpartland, R. A. McPherson, S. Mehlhase, A. Mehta, D. Melini, B. R. Mellado Garcia, A. H. Melo, F. Meloni, A. M. Mendes Jacques Da Costa, L. Meng, S. Menke, M. Mentink, E. Meoni, G. Mercado, S. Merianos, C. Merlassino, C. Meroni, J. Metcalfe, A. S. Mete, E. Meuser, C. Meyer, J-P. Meyer, Y. Miao, R. P. Middleton, M. Mihovilovic, L. Mijović, G. Mikenberg, M. Mikestikova, M. Mikuž, H. Mildner, A. Milic, D. W. Miller, E. H. Miller, L. S. Miller, A. Milov, D. A. Milstead, T. Min, A. A. Minaenko, I. A. Minashvili, A. I. Mincer, B. Mindur, M. Mineev, Y. Mino, L. M. Mir, M. Miralles Lopez, M. Mironova, M. C. Missio, A. Mitra, V. A. Mitsou, Y. Mitsumori, O. Miu, P. S. Miyagawa, T. Mkrtchyan, M. Mlinarevic, T. Mlinarevic, M. Mlynarikova, S. Mobius, M. H. Mohamed Farook, S. Mohapatra, S. Mohiuddin, G. Mokgatitswane, L. Moleri, U. Molinatti, L. G. Mollier, B. Mondal, S. Mondal, K. Mönig, E. Monnier, L. Monsonis Romero, J. Montejo Berlingen, A. Montella, M. Montella, F. Montereali, F. Monticelli, S. Monzani, A. Morancho Tarda, N. Morange, A. L. Moreira De Carvalho, M. Moreno Llácer, C. Moreno Martinez, J. M. Moreno Perez, P. Morettini, S. Morgenstern, M. Morii, M. Morinaga, M. Moritsu, F. Morodei, P. Moschovakos, B. Moser, M. Mosidze, T. Moskalets, P. Moskvitina, J. Moss, P. Moszkowicz, A. Moussa, Y. Moyal, H. Moyano Gomez, E. J. W. Moyse, T. G. Mroz, O. Mtintsilana, S. Muanza, M. Mucha, J. Mueller, R. Müller, G. A. Mullier, A. J. Mullin, J. J. Mullin, A. C. Mullins, A. E. Mulski, D. P. Mungo, D. Munoz Perez, F. J. Munoz Sanchez, W. J. Murray, M. Muškinja, C. Mwewa, A. G. Myagkov, A. J. Myers, G. Myers, M. Myska, B. P. Nachman, K. Nagai, K. Nagano, R. Nagasaka, J. L. Nagle, E. Nagy, A. M. Nairz, Y. Nakahama, K. Nakamura, K. Nakkalil, A. Nandi, H. Nanjo, E. A. Narayanan, Y. Narukawa, I. Naryshkin, L. Nasella, S. Nasri, C. Nass, G. Navarro, J. Navarro-Gonzalez, A. Nayaz, P. Y. Nechaeva, S. Nechaeva, F. Nechansky, L. Nedic, T. J. Neep, A. Negri, M. Negrini, C. Nellist, C. Nelson, K. Nelson, S. Nemecek, M. Nessi, M. S. Neubauer, J. Newell, P. R. Newman, Y. W. Y. Ng, B. Ngair, H. D. N. Nguyen, J. D. Nichols, R. B. Nickerson, R. Nicolaidou, J. Nielsen, M. Niemeyer, J. Niermann, N. Nikiforou, V. Nikolaenko, I. Nikolic-Audit, P. Nilsson, I. Ninca, G. Ninio, A. Nisati, R. Nisius, N. Nitika, J-E. Nitschke, E. K. Nkadimeng, T. Nobe, D. Noll, T. Nommensen, M. B. Norfolk, B. J. Norman, M. Noury, J. Novak, T. Novak, R. Novotny, L. Nozka, K. Ntekas, N. M. J. Nunes De Moura Junior, J. Ocariz, A. Ochi, I. Ochoa, S. Oerdek, J. T. Offermann, A. Ogrodnik, A. Oh, C. C. Ohm, H. Oide, M. L. Ojeda, Y. Okumura, L. F. Oleiro Seabra, I. Oleksiyuk, G. Oliveira Correa, D. Oliveira Damazio, J. L. Oliver, R. Omar, Ö. O. Öncel, A. P. O’Neill, A. Onofre, P. U. E. Onyisi, M. J. Oreglia, D. Orestano, R. Orlandini, R. S. Orr, L. M. Osojnak, Y. Osumi, G. Otero y Garzon, H. Otono, M. Ouchrif, F. Ould-Saada, T. Ovsiannikova, M. Owen, R. E. Owen, V. E. Ozcan, F. Ozturk, N. Ozturk, S. Ozturk, H. A. Pacey, K. Pachal, A. Pacheco Pages, C. Padilla Aranda, G. Padovano, S. Pagan Griso, G. Palacino, A. Palazzo, J. Pampel, J. Pan, T. Pan, D. K. Panchal, C. E. Pandini, J. G. Panduro Vazquez, H. D. Pandya, H. Pang, P. Pani, G. Panizzo, L. Panwar, L. Paolozzi, S. Parajuli, A. Paramonov, C. Paraskevopoulos, D. Paredes Hernandez, A. Pareti, K. R. Park, T. H. Park, F. Parodi, J. A. Parsons, U. Parzefall, B. Pascual Dias, L. Pascual Dominguez, E. Pasqualucci, S. Passaggio, F. Pastore, P. Patel, U. M. Patel, J. R. Pater, T. Pauly, F. Pauwels, C. I. Pazos, M. Pedersen, R. Pedro, S. V. Peleganchuk, O. Penc, E. A. Pender, S. Peng, G. D. Penn, K. E. Penski, M. Penzin, B. S. Peralva, A. P. Pereira Peixoto, L. Pereira Sanchez, D. V. Perepelitsa, G. Perera, E. Perez Codina, M. Perganti, H. Pernegger, S. Perrella, K. Peters, R. F. Y. Peters, B. A. Petersen, T. C. Petersen, E. Petit, V. Petousis, A. R. Petri, C. Petridou, T. Petru, A. Petrukhin, M. Pettee, A. Petukhov, K. Petukhova, R. Pezoa, L. Pezzotti, G. Pezzullo, L. Pfaffenbichler, A. J. Pfleger, T. M. Pham, T. Pham, P. W. Phillips, G. Piacquadio, E. Pianori, F. Piazza, R. Piegaia, D. Pietreanu, A. D. Pilkington, M. Pinamonti, J. L. Pinfold, B. C. Pinheiro Pereira, J. Pinol Bel, A. E. Pinto Pinoargote, L. Pintucci, K. M. Piper, A. Pirttikoski, D. A. Pizzi, L. Pizzimento, A. Plebani, M.-A. Pleier, V. Pleskot, E. Plotnikova, G. Poddar, R. Poettgen, L. Poggioli, S. Polacek, G. Polesello, A. Poley, A. Polini, C. S. Pollard, Z. B. Pollock, E. Pompa Pacchi, N. I. Pond, D. Ponomarenko, L. Pontecorvo, S. Popa, G. A. Popeneciu, A. Poreba, D. M. Portillo Quintero, S. Pospisil, M. A. Postill, P. Postolache, K. Potamianos, P. A. Potepa, I. N. Potrap, C. J. Potter, H. Potti, J. Poveda, M. E. Pozo Astigarraga, R. Pozzi, A. Prades Ibanez, S. R. Pradhan, J. Pretel, D. Price, M. Primavera, L. Primomo, M. A. Principe Martin, R. Privara, T. Procter, M. L. Proffitt, N. Proklova, K. Prokofiev, G. Proto, J. Proudfoot, M. Przybycien, W. W. Przygoda, A. Psallidas, J. E. Puddefoot, D. Pudzha, H. I. Purnell, D. Pyatiizbyantseva, J. Qian, R. Qian, D. Qichen, Y. Qin, T. Qiu, A. Quadt, M. Queitsch-Maitland, G. Quetant, R. P. Quinn, G. Rabanal Bolanos, D. Rafanoharana, F. Raffaeli, F. Ragusa, J. L. Rainbolt, J. A. Raine, S. Rajagopalan, E. Ramakoti, L. Rambelli, I. A. Ramirez-Berend, K. Ran, D. S. Rankin, N. P. Rapheeha, H. Rasheed, D. F. Rassloff, A. Rastogi, S. Rave, S. Ravera, B. Ravina, I. Ravinovich, M. Raymond, A. L. Read, N. P. Readioff, D. M. Rebuzzi, A. S. Reed, K. Reeves, J. A. Reidelsturz, D. Reikher, A. Rej, C. Rembser, H. Ren, M. Renda, F. Renner, A. G. Rennie, A. L. Rescia, S. Resconi, M. Ressegotti, S. Rettie, W. F. Rettie, M. M. Revering, E. Reynolds, O. L. Rezanova, P. Reznicek, H. Riani, N. Ribaric, B. Ricci, E. Ricci, R. Richter, S. Richter, E. Richter-Was, M. Ridel, S. Ridouani, P. Rieck, P. Riedler, E. M. Riefel, J. O. Rieger, M. Rijssenbeek, M. Rimoldi, L. Rinaldi, P. Rincke, G. Ripellino, I. Riu, J. C. Rivera Vergara, F. Rizatdinova, E. Rizvi, B. R. Roberts, S. S. Roberts, D. Robinson, M. Robles Manzano, A. Robson, A. Rocchi, C. Roda, S. Rodriguez Bosca, Y. Rodriguez Garcia, A. M. Rodríguez Vera, S. Roe, J. T. Roemer, O. Røhne, R. A. Rojas, C. P. A. Roland, A. Romaniouk, E. Romano, M. Romano, A. C. Romero Hernandez, N. Rompotis, L. Roos, S. Rosati, B. J. Rosser, E. Rossi, E. Rossi, L. P. Rossi, L. Rossini, R. Rosten, M. Rotaru, B. Rottler, D. Rousseau, D. Rousso, S. Roy-Garand, A. Rozanov, Z. M. A. Rozario, Y. Rozen, A. Rubio Jimenez, V. H. Ruelas Rivera, T. A. Ruggeri, A. Ruggiero, A. Ruiz-Martinez, A. Rummler, Z. Rurikova, N. A. Rusakovich, S. Ruscelli, H. L. Russell, G. Russo, J. P. Rutherfoord, S. Rutherford Colmenares, M. Rybar, P. Rybczynski, A. Ryzhov, J. A. Sabater Iglesias, H. F-W. Sadrozinski, F. Safai Tehrani, S. Saha, M. Sahinsoy, B. Sahoo, A. Saibel, B. T. Saifuddin, M. Saimpert, G. T. Saito, M. Saito, T. Saito, A. Sala, A. Salnikov, J. Salt, A. Salvador Salas, F. Salvatore, A. Salzburger, D. Sammel, E. Sampson, D. Sampsonidis, D. Sampsonidou, J. Sánchez, V. Sanchez Sebastian, H. Sandaker, C. O. Sander, J. A. Sandesara, M. Sandhoff, C. Sandoval, L. Sanfilippo, D. P. C. Sankey, T. Sano, A. Sansoni, M. Santana Queiroz, L. Santi, C. Santoni, H. Santos, A. Santra, E. Sanzani, K. A. Saoucha, J. G. Saraiva, J. Sardain, O. Sasaki, K. Sato, C. Sauer, E. Sauvan, P. Savard, R. Sawada, C. Sawyer, L. Sawyer, C. Sbarra, A. Sbrizzi, T. Scanlon, J. Schaarschmidt, U. Schäfer, A. C. Schaffer, D. Schaile, R. D. Schamberger, C. Scharf, M. M. Schefer, V. A. Schegelsky, D. Scheirich, M. Schernau, C. Scheulen, C. Schiavi, M. Schioppa, B. Schlag, S. Schlenker, J. Schmeing, E. Schmidt, M. A. Schmidt, K. Schmieden, C. Schmitt, N. Schmitt, S. Schmitt, N. A. Schneider, L. Schoeffel, A. Schoening, P. G. Scholer, E. Schopf, M. Schott, S. Schramm, T. Schroer, H-C. Schultz-Coulon, M. Schumacher, B. A. Schumm, Ph. Schune, H. R. Schwartz, A. Schwartzman, T. A. Schwarz, Ph. Schwemling, R. Schwienhorst, F. G. Sciacca, A. Sciandra, G. Sciolla, F. Scuri, C. D. Sebastiani, K. Sedlaczek, S. C. Seidel, A. Seiden, B. D. Seidlitz, C. Seitz, J. M. Seixas, G. Sekhniaidze, L. Selem, N. Semprini-Cesari, A. Semushin, D. Sengupta, V. Senthilkumar, L. Serin, M. Sessa, H. Severini, F. Sforza, A. Sfyrla, Q. Sha, E. Shabalina, H. Shaddix, A. H. Shah, R. Shaheen, J. D. Shahinian, M. Shamim, L. Y. Shan, M. Shapiro, A. Sharma, A. S. Sharma, P. Sharma, P. B. Shatalov, K. Shaw, S. M. Shaw, Q. Shen, D. J. Sheppard, P. Sherwood, L. Shi, X. Shi, S. Shimizu, C. O. Shimmin, I. P. J. Shipsey, S. Shirabe, M. Shiyakova, M. J. Shochet, D. R. Shope, B. Shrestha, S. Shrestha, I. Shreyber, M. J. Shroff, P. Sicho, A. M. Sickles, E. Sideras Haddad, A. C. Sidley, A. Sidoti, F. Siegert, Dj. Sijacki, F. Sili, J. M. Silva, I. Silva Ferreira, M. V. Silva Oliveira, S. B. Silverstein, S. Simion, R. Simoniello, E. L. Simpson, H. Simpson, L. R. Simpson, S. Simsek, S. Sindhu, P. Sinervo, S. N. Singh, S. Singh, S. Sinha, S. Sinha, M. Sioli, K. Sioulas, I. Siral, E. Sitnikova, J. Sjölin, A. Skaf, E. Skorda, P. Skubic, M. Slawinska, I. Slazyk, I. Sliusar, V. Smakhtin, B. H. Smart, S. Yu. Smirnov, Y. Smirnov, L. N. Smirnova, O. Smirnova, A. C. Smith, D. R. Smith, J. L. Smith, M. B. Smith, R. Smith, H. Smitmanns, M. Smizanska, K. Smolek, P. Smolyanskiy, A. A. Snesarev, H. L. Snoek, S. Snyder, R. Sobie, A. Soffer, C. A. Solans Sanchez, E. Yu. Soldatov, U. Soldevila, A. A. Solodkov, S. Solomon, A. Soloshenko, K. Solovieva, O. V. Solovyanov, P. Sommer, A. Sonay, A. Sopczak, A. L. Sopio, F. Sopkova, J. D. Sorenson, I. R. Sotarriva Alvarez, V. Sothilingam, O. J. Soto Sandoval, S. Sottocornola, R. Soualah, Z. Soumaimi, D. South, N. Soybelman, S. Spagnolo, M. Spalla, D. Sperlich, B. Spisso, D. P. Spiteri, L. Splendori, M. Spousta, E. J. Staats, R. Stamen, E. Stanecka, W. Stanek-Maslouska, M. V. Stange, B. Stanislaus, M. M. Stanitzki, B. Stapf, E. A. Starchenko, G. H. Stark, J. Stark, P. Staroba, P. Starovoitov, R. Staszewski, C. Stauch, G. Stavropoulos, A. Stefl, A. Stein, P. Steinberg, B. Stelzer, H. J. Stelzer, O. Stelzer, H. Stenzel, T. J. Stevenson, G. A. Stewart, J. R. Stewart, M. C. Stockton, G. Stoicea, M. Stolarski, S. Stonjek, A. Straessner, J. Strandberg, S. Strandberg, M. Stratmann, M. Strauss, T. Strebler, P. Strizenec, R. Ströhmer, D. M. Strom, R. Stroynowski, A. Strubig, S. A. Stucci, B. Stugu, J. Stupak, N. A. Styles, D. Su, S. Su, X. Su, D. Suchy, K. Sugizaki, V. V. Sulin, M. J. Sullivan, D. M. S. Sultan, L. Sultanaliyeva, S. Sultansoy, S. Sun, W. Sun, O. Sunneborn Gudnadottir, N. Sur, M. R. Sutton, H. Suzuki, M. Svatos, P. N. Swallow, M. Swiatlowski, T. Swirski, A. Swoboda, I. Sykora, M. Sykora, T. Sykora, D. Ta, K. Tackmann, A. Taffard, R. Tafirout, Y. Takubo, M. Talby, A. A. Talyshev, K. C. Tam, N. M. Tamir, A. Tanaka, J. Tanaka, R. Tanaka, M. Tanasini, Z. Tao, S. Tapia Araya, S. Tapprogge, A. Tarek Abouelfadl Mohamed, S. Tarem, K. Tariq, G. Tarna, G. F. Tartarelli, M. J. Tartarin, P. Tas, M. Tasevsky, E. Tassi, A. C. Tate, G. Tateno, Y. Tayalati, G. N. Taylor, W. Taylor, A. S. Tegetmeier, P. Teixeira-Dias, J. J. Teoh, K. Terashi, J. Terron, S. Terzo, M. Testa, R. J. Teuscher, A. Thaler, O. Theiner, T. Theveneaux-Pelzer, D. W. Thomas, J. P. Thomas, E. A. Thompson, P. D. Thompson, E. Thomson, R. E. Thornberry, C. Tian, Y. Tian, V. Tikhomirov, Yu. A. Tikhonov, S. Timoshenko, D. Timoshyn, E. X. L. Ting, P. Tipton, A. Tishelman-Charny, K. Todome, S. Todorova-Nova, L. Toffolin, M. Togawa, J. Tojo, S. Tokár, O. Toldaiev, G. Tolkachev, M. Tomoto, L. Tompkins, E. Torrence, H. Torres, E. Torró Pastor, M. Toscani, C. Tosciri, M. Tost, D. R. Tovey, T. Trefzger, P. M. Tricarico, A. Tricoli, I. M. Trigger, S. Trincaz-Duvoid, D. A. Trischuk, A. Tropina, L. Truong, M. Trzebinski, A. Trzupek, F. Tsai, M. Tsai, A. Tsiamis, P. V. Tsiareshka, S. Tsigaridas, A. Tsirigotis, V. Tsiskaridze, E. G. Tskhadadze, M. Tsopoulou, Y. Tsujikawa, I. I. Tsukerman, V. Tsulaia, S. Tsuno, K. Tsuri, D. Tsybychev, Y. Tu, A. Tudorache, V. Tudorache, S. B. Tuncay, S. Turchikhin, I. Turk Cakir, R. Turra, T. Turtuvshin, P. M. Tuts, S. Tzamarias, E. Tzovara, Y. Uematsu, F. Ukegawa, P. A. Ulloa Poblete, E. N. Umaka, G. Unal, A. Undrus, G. Unel, J. Urban, P. Urrejola, G. Usai, R. Ushioda, M. Usman, F. Ustuner, Z. Uysal, V. Vacek, B. Vachon, T. Vafeiadis, A. Vaitkus, C. Valderanis, E. Valdes Santurio, M. Valente, S. Valentinetti, A. Valero, E. Valiente Moreno, A. Vallier, J. A. Valls Ferrer, D. R. Van Arneman, A. Van Der Graaf, H. Z. Van Der Schyf, P. Van Gemmeren, M. Van Rijnbach, S. Van Stroud, I. Van Vulpen, P. Vana, M. Vanadia, U. M. Vande Voorde, W. Vandelli, E. R. Vandewall, D. Vannicola, L. Vannoli, R. Vari, M. Varma, E. W. Varnes, C. Varni, D. Varouchas, L. Varriale, K. E. Varvell, M. E. Vasile, L. Vaslin, M. D. Vassilev, A. Vasyukov, L. M. Vaughan, R. Vavricka, T. Vazquez Schroeder, J. Veatch, V. Vecchio, M. J. Veen, I. Veliscek, I. Velkovska, L. M. Veloce, F. Veloso, S. Veneziano, A. Ventura, A. Verbytskyi, M. Verducci, C. Vergis, M. Verissimo De Araujo, W. Verkerke, J. C. Vermeulen, C. Vernieri, M. Vessella, M. C. Vetterli, A. Vgenopoulos, N. Viaux Maira, T. Vickey, O. E. Vickey Boeriu, G. H. A. Viehhauser, L. Vigani, M. Vigl, M. Villa, M. Villaplana Perez, E. M. Villhauer, E. Vilucchi, M. Vincent, M. G. Vincter, A. Visibile, C. Vittori, I. Vivarelli, E. Voevodina, F. Vogel, J. C. Voigt, P. Vokac, Yu. Volkotrub, L. Vomberg, E. Von Toerne, B. Vormwald, K. Vorobev, M. Vos, K. Voss, M. Vozak, L. Vozdecky, N. Vranjes, M. Vranjes Milosavljevic, M. Vreeswijk, N. K. Vu, R. Vuillermet, O. Vujinovic, I. Vukotic, I. K. Vyas, J. F. Wack, S. Wada, C. Wagner, J. M. Wagner, W. Wagner, S. Wahdan, H. Wahlberg, C. H. Waits, J. Walder, R. Walker, K. Walkingshaw Pass, W. Walkowiak, A. Wall, E. J. Wallin, T. Wamorkar, K. Wandall-Christensen, A. Wang, A. Z. Wang, C. Wang, C. Wang, H. Wang, J. Wang, P. Wang, P. Wang, R. Wang, R. Wang, S. M. Wang, S. Wang, T. Wang, W. T. Wang, W. Wang, X. Wang, X. Wang, X. Wang, Y. Wang, Y. Wang, Z. Wang, Z. Wang, C. Wanotayaroj, A. Warburton, A. L. Warnerbring, N. Warrack, S. Waterhouse, A. T. Watson, H. Watson, M. F. Watson, E. Watton, G. Watts, B. M. Waugh, J. M. Webb, C. Weber, H. A. Weber, M. S. Weber, S. M. Weber, C. Wei, Y. Wei, A. R. Weidberg, E. J. Weik, J. Weingarten, C. Weiser, C. J. Wells, T. Wenaus, B. Wendland, T. Wengler, N. S. Wenke, N. Wermes, M. Wessels, A. M. Wharton, A. S. White, A. White, M. J. White, D. Whiteson, L. Wickremasinghe, W. Wiedenmann, M. Wielers, R. Wierda, C. Wiglesworth, H. G. Wilkens, J. J. H. Wilkinson, D. M. Williams, H. H. Williams, S. Williams, S. Willocq, B. J. Wilson, D. J. Wilson, P. J. Windischhofer, F. I. Winkel, F. Winklmeier, B. T. Winter, M. Wittgen, M. Wobisch, T. Wojtkowski, Z. Wolffs, J. Wollrath, M. W. Wolter, H. Wolters, M. C. Wong, E. L. Woodward, S. D. Worm, B. K. Wosiek, K. W. Woźniak, S. Wozniewski, K. Wraight, C. Wu, C. Wu, J. Wu, M. Wu, M. Wu, S. L. Wu, S. Wu, X. Wu, Y. Wu, Z. Wu, Z. Wu, J. Wuerzinger, T. R. Wyatt, B. M. Wynne, S. Xella, L. Xia, M. Xia, M. Xie, A. Xiong, J. Xiong, D. Xu, H. Xu, L. Xu, R. Xu, T. Xu, Y. Xu, Z. Xu, R. Xue, B. Yabsley, S. Yacoob, Y. Yamaguchi, E. Yamashita, H. Yamauchi, T. Yamazaki, Y. Yamazaki, S. Yan, Z. Yan, H. J. Yang, H. T. Yang, S. Yang, T. Yang, X. Yang, X. Yang, Y. Yang, Y. Yang, W-M. Yao, C. L. Yardley, J. Ye, S. Ye, X. Ye, Y. Yeh, I. Yeletskikh, B. Yeo, M. R. Yexley, T. P. Yildirim, K. Yorita, C. J. S. Young, C. Young, N. D. Young, Y. Yu, J. Yuan, M. Yuan, R. Yuan, L. Yue, M. Zaazoua, B. Zabinski, I. Zahir, A. Zaio, Z. K. Zak, T. Zakareishvili, S. Zambito, J. A. Zamora Saa, J. Zang, R. Zanzottera, O. Zaplatilek, C. Zeitnitz, H. Zeng, J. C. Zeng, D. T. Zenger, O. Zenin, T. Ženiš, S. Zenz, D. Zerwas, M. Zhai, D. F. Zhang, G. Zhang, J. Zhang, J. Zhang, K. Zhang, L. Zhang, L. Zhang, P. Zhang, R. Zhang, S. Zhang, T. Zhang, Y. Zhang, Y. Zhang, Y. Zhang, Y. Zhang, Z. Zhang, Z. Zhang, H. Zhao, T. Zhao, Y. Zhao, Z. Zhao, A. Zhemchugov, J. Zheng, K. Zheng, X. Zheng, Z. Zheng, D. Zhong, B. Zhou, H. Zhou, N. Zhou, Y. Zhou, Y. Zhou, Y. Zhou, C. G. Zhu, J. Zhu, X. Zhu, Y. Zhu, Y. Zhu, X. Zhuang, K. Zhukov, N. I. Zimine, J. Zinsser, M. Ziolkowski, L. Živković, A. Zoccoli, K. Zoch, A. Zografos, T. G. Zorbas, O. Zormpa, L. Zwalinski

**Affiliations:** 1 CPPM, Aix-Marseille Université, CNRS/IN2P3, Marseille, France; 2 Department for Physics and Technology, University of Bergen, Bergen, Norway; 3 Homer L. Dodge Department of Physics and Astronomy, University of Oklahoma, Norman, OK USA; 4 New York University Abu Dhabi, Abu Dhabi, United Arab Emirates; 5 II. Physikalisches Institut, Georg-August-Universität Göttingen, Göttingen, Germany; 6 Fakultät Physik, Technische Universität Dortmund, Dortmund, Germany; 7 Physics Department, Brookhaven National Laboratory, Upton, NY USA; 8 Physics Department, Southern Methodist University, Dallas, TX USA; 9 Faculté des sciences, Université Mohammed V, Rabat, Morocco; 10 Raymond and Beverly Sackler School of Physics and Astronomy, Tel Aviv University, Tel Aviv, Israel; 11 Department of Physics, New York University, New York, NY USA; 12 INFN Gruppo Collegato di Udine, Sezione di Trieste, Udine, Italy; 13 ICTP, Trieste, Italy; 14 Kirchhoff-Institut für Physik, Ruprecht-Karls-Universität Heidelberg, Heidelberg, Germany; 15 LAPP, Université Savoie Mont Blanc, CNRS/IN2P3, Annecy, France; 16 Faculty of Physics and Applied Computer Science, AGH University of Krakow, Krakow, Poland; 17 SLAC National Accelerator Laboratory, Stanford, CA USA; 18 School of Physics and Astronomy, University of Manchester, Manchester, UK; 19 Department of Physics, Northern Illinois University, DeKalb, IL USA; 20 Department of Physics, Istanbul University, Istanbul, Türkiye; 21 Particle Physics Department, Rutherford Appleton Laboratory, Didcot, UK; 22 Santa Cruz Institute for Particle Physics, University of California Santa Cruz, Santa Cruz, CA USA; 23 Enrico Fermi Institute, University of Chicago, Chicago, IL USA; 24 Institut de Física d’Altes Energies (IFAE), Barcelona Institute of Science and Technology, Barcelona, Spain; 25 Institut für Physik, Universität Mainz, Mainz, Germany; 26 Department of Physics, Alexandru Ioan Cuza University of Iasi, Iasi, Romania; 27 Affiliated with an international laboratory covered by a cooperation agreement with CERN, Geneva, Switzerland; 28 Department of Physics, Royal Holloway University of London, Egham, UK; 29 School of Physics, Zhengzhou University, Zhengzhou, China; 30 INFN Sezione di Roma Tor Vergata, Rome, Italy; 31 Dipartimento di Fisica, Università di Roma Tor Vergata, Rome, Italy; 32 Instituto de Física Corpuscular (IFIC), Centro Mixto Universidad de Valencia - CSIC, Valencia, Spain; 33 Faculté des Sciences Ain Chock, Université Hassan II de Casablanca, Casablanca, Morocco; 34 Fysiska institutionen, Lunds universitet, Lund, Sweden; 35 Departments of Physics and Astronomy, Stony Brook University, Stony Brook, NY USA; 36 Waseda University, Tokyo, Japan; 37 Physikalisches Institut, Universität Bonn, Bonn, Germany; 38 Department of Physics, Bogazici University, Istanbul, Türkiye; 39 INFN Sezione di Bologna, Bologna, Italy; 40 Department of Physics and Astronomy, University of Victoria, Victoria, BC Canada; 41 Albert Einstein Center for Fundamental Physics and Laboratory for High Energy Physics, University of Bern, Bern, Switzerland; 42 INFN e Laboratori Nazionali di Frascati, Frascati, Italy; 43 LPSC, Université Grenoble Alpes, CNRS/IN2P3, Grenoble INP, Grenoble, France; 44 SUPA - School of Physics and Astronomy, University of Edinburgh, Edinburgh, UK; 45 Department of Physics, Oklahoma State University, Stillwater, OK USA; 46 CERN, Geneva, Switzerland; 47 Horia Hulubei National Institute of Physics and Nuclear Engineering, Bucharest, Romania; 48 Physics Department, National Technical University of Athens, Zografou, Greece; 49 Département de Physique Nucléaire et Corpusculaire, Université de Genève, Geneva, Switzerland; 50 Department of Physics, University of Warwick, Coventry, UK; 51 Czech Technical University in Prague, Prague, Czech Republic; 52 Physics Department, Lancaster University, Lancaster, UK; 53 California State University, Fresno, CA USA; 54 Oliver Lodge Laboratory, University of Liverpool, Liverpool, UK; 55 Department of Mechanical Engineering Science, University of Johannesburg, Johannesburg, South Africa; 56 INFN Sezione di Milano, Milan, Italy; 57 IJCLab, Université Paris-Saclay, CNRS/IN2P3, Orsay, France; 58 Department of Physics and Astronomy, University of Sussex, Brighton, UK; 59 School of Physics and Astronomy, University of Birmingham, Birmingham, UK; 60 INFN Sezione di Napoli, Naples, Italy; 61 Dipartimento di Fisica, Università di Napoli, Naples, Italy; 62 Instituto de Física La Plata, Universidad Nacional de La Plata and CONICET, La Plata, Argentina; 63 Department of Physics, University of Washington, Seattle, WA USA; 64 Universidade Federal do Rio De Janeiro COPPE/EE/IF, Rio de Janeiro, Brazil; 65 Department of Physics, McGill University, Montreal, QC Canada; 66 Fakultät für Physik, Ludwig-Maximilians-Universität München, München, Germany; 67 LPNHE, Sorbonne Université, Université Paris Cité, CNRS/IN2P3, Paris, France; 68 Department of Physics, University of Michigan, Ann Arbor, MI USA; 69 Physikalisches Institut, Albert-Ludwigs-Universität Freiburg, Freiburg, Germany; 70 Department of Physics, University of Toronto, Toronto, ON Canada; 71 Laboratório de Instrumentação e Física Experimental de Partículas - LIP, Lisbon, Portugal; 72 Department of Physics, Aristotle University of Thessaloniki, Thessaloniki, Greece; 73 KEK, High Energy Accelerator Research Organization, Tsukuba, Japan; 74 Department of Physics and Astronomy, University of Sheffield, Sheffield, UK; 75 Department of Physics, University of Texas at Austin, Austin, TX USA; 76 SUPA - School of Physics and Astronomy, University of Glasgow, Glasgow, UK; 77 Dipartimento di Fisica, Università di Milano, Milan, Italy; 78 Physics Department, National and Kapodistrian University of Athens, Athens, Greece; 79 Nevis Laboratory, Columbia University, Irvington, NY USA; 80 INFN Sezione di Pisa, Pisa, Italy; 81 INFN Sezione di Roma, Rome, Italy; 82 International Center for Elementary Particle Physics and Department of Physics, University of Tokyo, Tokyo, Japan; 83 Deutsches Elektronen-Synchrotron DESY, Hamburg and Zeuthen, Germany; 84 Universidad de Buenos Aires, Facultad de Ciencias Exactas y Naturales, Departamento de Física, y CONICET, Instituto de Física de Buenos Aires (IFIBA), Buenos Aires, Argentina; 85 Department of Physics, Brandeis University, Waltham, MA USA; 86 Institute of Nuclear Physics Polish Academy of Sciences, Krakow, Poland; 87 Department of Physics, Duke University, Durham, NC USA; 88 Group of Particle Physics, University of Montreal, Montreal, QC Canada; 89 Dipartimento di Fisica, Sapienza Università di Roma, Rome, Italy; 90 Graduate School of Science and Kobayashi-Maskawa Institute, Nagoya University, Nagoya, Japan; 91 Ochanomizu University, Bunkyo-ku, Tokyo Japan; 92 Yerevan Physics Institute, Yerevan, Armenia; 93 Faculty of Mathematics, Physics and Informatics, Comenius University, Bratislava, Slovakia; 94 Department of Physics and Astronomy, University of California Irvine, Irvine, CA USA; 95 Department of Physics, University of Cape Town, Cape Town, South Africa; 96 Institute of Applied Physics, Mohammed VI Polytechnic University, Ben Guerir, Morocco; 97 Department of Physics, University of Pennsylvania, Philadelphia, PA USA; 98 LPC, Université Clermont Auvergne, CNRS/IN2P3, Clermont-Ferrand, France; 99 Department of Subnuclear Physics, Institute of Experimental Physics of the Slovak Academy of Sciences, Kosice, Slovak Republic; 100 IRFU, CEA, Université Paris-Saclay, Gif-sur-Yvette, France; 101 Department of Physics, Carleton University, Ottawa, ON Canada; 102 Institut für Kern- und Teilchenphysik, Technische Universität Dresden, Dresden, Germany; 103 Institut für Physik, Humboldt Universität zu Berlin, Berlin, Germany; 104 Institute for Fundamental Science, University of Oregon, Eugene, OR USA; 105 Department of Physics and Astronomy, Queen Mary University of London, London, UK; 106 Department of Physics, Yale University, New Haven, CT USA; 107 Institute of Physics, University of Belgrade, Belgrade, Serbia; 108 Department of Physics, University of Texas at Arlington, Arlington, TX USA; 109 Affiliated with an institute formerly covered by a cooperation agreement with CERN, Geneva, Switzerland; 110 Dipartimento di Fisica e Astronomia A. Righi, Università di Bologna, Bologna, Italy; 111 Cavendish Laboratory, University of Cambridge, Cambridge, UK; 112 United Arab Emirates University, Al Ain, United Arab Emirates; 113 Department of Physics and Astronomy, University of Pittsburgh, Pittsburgh, PA USA; 114 School of Physics, University of Melbourne, Melbourne, VIC Australia; 115 University of California, Berkeley, CA USA; 116 Nikhef National Institute for Subatomic Physics and University of Amsterdam, Amsterdam, The Netherlands; 117 Max-Planck-Institut für Physik (Werner-Heisenberg-Institut), München, Germany; 118 Charles University, Faculty of Mathematics and Physics, Prague, Czech Republic; 119 Department of Modern Physics and State Key Laboratory of Particle Detection and Electronics, University of Science and Technology of China, Hefei, China; 120 Department of Physics, Oxford University, Oxford, UK; 121 Department of Physics and Astronomy, University College London, London, UK; 122 Departamento de Física Teorica C-15 and CIAFF, Universidad Autónoma de Madrid, Madrid, Spain; 123 Institute of High Energy Physics, Chinese Academy of Sciences, Beijing, China; 124 TRIUMF, Vancouver, BC Canada; 125 Department of Physics, University of British Columbia, Vancouver, BC Canada; 126 Universität Innsbruck, Department of Astro and Particle Physics, Innsbruck, Austria; 127 Department of Physics and Astronomy, Tufts University, Medford, MA USA; 128 Istinye University, Sariyer, Istanbul Türkiye; 129 Rio de Janeiro State University, Rio de Janeiro, Brazil; 130 Department of Physics, Indiana University, Bloomington, IN USA; 131 Laboratory for Particle Physics and Cosmology, Harvard University, Cambridge, MA USA; 132 Department of Physics and Astronomy, University of Uppsala, Uppsala, Sweden; 133 Physics Division, Lawrence Berkeley National Laboratory, Berkeley, CA USA; 134 APC, Université Paris Cité, CNRS/IN2P3, Paris, France; 135 INFN Sezione di Roma Tre, Rome, Italy; 136 Fakultät für Mathematik und Naturwissenschaften, Fachgruppe Physik, Bergische Universität Wuppertal, Wuppertal, Germany; 137 Department of Physics Engineering, Gaziantep University, Gaziantep, Türkiye; 138 Department of Particle Physics and Astrophysics, Weizmann Institute of Science, Rehovot, Israel; 139 Department Physik, Universität Siegen, Siegen, Germany; 140 Dipartimento di Fisica, Università di Genova, Genoa, Italy; 141 INFN Sezione di Genova, Genoa, Italy; 142 Fakultät für Physik und Astronomie, Julius-Maximilians-Universität Würzburg, Würzburg, Germany; 143 Ohio State University, Columbus, OH USA; 144 Department of Physics, University of Massachusetts, Amherst, MA USA; 145 INFN-TIFPA, Trento, Italy; 146 Università degli Studi di Trento, Trento, Italy; 147 Department of Physics and Astronomy, Michigan State University, East Lansing, MI USA; 148 Department of Physics and Astronomy, York University, Toronto, ON Canada; 149 Department of Physics, Stockholm University, Stockholm, Sweden; 150 Oskar Klein Centre, Stockholm, Sweden; 151 L2IT, Université de Toulouse, CNRS/IN2P3, UPS, Toulouse, France; 152 Department of Physics, University of Illinois, Urbana, IL USA; 153 Department of Physics, Simon Fraser University, Burnaby, BC Canada; 154 Department of Physics, Boston University, Boston, MA USA; 155 II. Physikalisches Institut, Justus-Liebig-Universität Giessen, Giessen, Germany; 156 University of Chinese Academy of Science (UCAS), Beijing, China; 157 Department of Physics, Nanjing University, Nanjing, China; 158 Department of Physics, Ankara University, Ankara, Türkiye; 159 Niels Bohr Institute, University of Copenhagen, Copenhagen, Denmark; 160 Dipartimento di Fisica, Università della Calabria, Rende, Italy; 161 INFN Gruppo Collegato di Cosenza, Laboratori Nazionali di Frascati, Frascati, Italy; 162 Institute for Mathematics, Astrophysics and Particle Physics, Radboud University/Nikhef, Nijmegen, The Netherlands; 163 Departamento de Física, Universidad Técnica Federico Santa María, Valparaíso, Chile; 164 INFN Sezione di Pavia, Pavia, Italy; 165 Dipartimento di Fisica, Università di Pavia, Pavia, Italy; 166 Departamento de Física, Escola de Ciências, Universidade do Minho, Braga, Portugal; 167 Department of Physics, University of Oslo, Oslo, Norway; 168 Departamento de Física, Universidad Nacional de Colombia, Bogotá, Colombia; 169 Joint Laboratory of Optics, Palacký University, Olomouc, Czech Republic; 170 Departamento de Engenharia Elétrica, Universidade Federal de Juiz de Fora (UFJF), Juiz de Fora, Brazil; 171 Dipartimento di Fisica E. Fermi, Università di Pisa, Pisa, Italy; 172 High Energy Physics Institute, Tbilisi State University, Tbilisi, Georgia; 173 High Energy Physics Division, Argonne National Laboratory, Lemont, IL USA; 174 State Key Laboratory of Dark Matter Physics, School of Physics and Astronomy, Shanghai Jiao Tong University, Key Laboratory for Particle Astrophysics and Cosmology (MOE), SKLPPC, Shanghai, China; 175 Faculty of Science, Kyoto University, Kyoto, Japan; 176 Physics Department, Tsinghua University, Beijing, China; 177 Department of Physics, University of Wisconsin, Madison, WI USA; 178 Department of Physics, Chinese University of Hong Kong, Shatin, N.T., Hong Kong, China; 179 Department of Physics, University of Arizona, Tucson, AZ USA; 180 Department of Physics, National Tsing Hua University, Hsinchu, Taiwan; 181 INFN Sezione di Lecce, Lecce, Italy; 182 Institute of Physics of the Czech Academy of Sciences, Prague, Czech Republic; 183 Department of Experimental Particle Physics, Jožef Stefan Institute and Department of Physics, University of Ljubljana, Ljubljana, Slovenia; 184 Dipartimento Politecnico di Ingegneria e Architettura, Università di Udine, Udine, Italy; 185 Dipartimento di Matematica e Fisica, Università del Salento, Lecce, Italy; 186 Departamento de Fisica, Instituto Superior Técnico, Universidade de Lisboa, Lisbon, Portugal; 187 Institute of Physics, Academia Sinica, Taipei, Taiwan; 188 Department of Physics, University of Adelaide, Adelaide, SA Australia; 189 Dipartimento di Matematica e Fisica, Università Roma Tre, Rome, Italy; 190 Departamento de Física, Pontificia Universidad Católica de Chile, Santiago, Chile; 191 Millennium Institute for Subatomic physics at high energy frontier (SAPHIR), Santiago, Chile; 192 Department of Physics, Royal Institute of Technology, Stockholm, Sweden; 193 Physikalisches Institut, Ruprecht-Karls-Universität Heidelberg, Heidelberg, Germany; 194 Federal University of Bahia, Salvador, Bahia Brazil; 195 Faculty of Physics, University of Bucharest, Bucharest, Romania; 196 Faculté des Sciences, Université Ibn-Tofail, Kénitra, Morocco; 197 Departamento de Física, Faculdade de Ciências, Universidade de Lisboa, Lisbon, Portugal; 198 Department of Physics, University of Hong Kong, Hong Kong, China; 199 Louisiana Tech University, Ruston, LA USA; 200 Institute of Frontier and Interdisciplinary Science and Key Laboratory of Particle Physics and Particle Irradiation (MOE), Shandong University, Qingdao, China; 201 Centro Nacional de Microelectrónica (IMB-CNM-CSIC), Barcelona, Spain; 202 Departamento de Física, Universidade de Coimbra, Coimbra, Portugal; 203 National Institute of Physics, University of the Philippines Diliman (Philippines), Quezon City, Philippines; 204 University of Sharjah, Sharjah, United Arab Emirates; 205 Department of Physics and Astronomy, University of New Mexico, Albuquerque, NM USA; 206 National Centre for Scientific Research “Demokritos”, Agia Paraskevi, Greece; 207 Marian Smoluchowski Institute of Physics, Jagiellonian University, Krakow, Poland; 208 Department of Physics, University of Alberta, Edmonton, AB Canada; 209 University of Georgia, Tbilisi, Georgia; 210 West University in Timisoara, Timisoara, Romania; 211 School of Science, Shenzhen Campus of Sun Yat-sen University, Guangzhou, China; 212 Department of Physics, Shinshu University, Nagano, Japan; 213 Graduate School of Science, University of Osaka, Osaka, Japan; 214 Division of Physics and Tomonaga Center for the History of the Universe, Faculty of Pure and Applied Sciences, University of Tsukuba, Tsukuba, Japan; 215 National University of Science and Technology Politechnica, Bucharest, Romania; 216 Institute of Physics, Azerbaijan Academy of Sciences, Baku, Azerbaijan; 217 Instituto de Investigación Multidisciplinario en Ciencia y Tecnología, y Departamento de Física, Universidad de La Serena, La Serena, Chile; 218 E. Andronikashvili Institute of Physics, Iv. Javakhishvili Tbilisi State University, Tbilisi, Georgia; 219 Department of Physics, Institute of Science, Tokyo, Japan; 220 Instituto de Alta Investigación, Universidad de Tarapacá, Arica, Chile; 221 Department of Physics, Technion, Israel Institute of Technology, Haifa, Israel; 222 School of Physics, University of the Witwatersrand, Johannesburg, South Africa; 223 Department of Physics and Astronomy, Iowa State University, Ames, IA USA; 224 Department of Physics, Universidad Andres Bello, Santiago, Chile; 225 Graduate School of Science, Kobe University, Kobe, Japan; 226 Instituto de Física, Universidade de São Paulo, São Paulo, Brazil; 227 State Key Laboratory of Dark Matter Physics, Tsung-Dao Lee Institute, Shanghai Jiao Tong University, Shanghai, China; 228 Department of Physics and Institute for Advanced Study, Hong Kong University of Science and Technology, Clear Water Bay, Kowloon, Hong Kong, China; 229 Centro de Física Nuclear da Universidade de Lisboa, Lisbon, Portugal; 230 University of Iowa, Iowa City, IA USA; 231 Research Center for Advanced Particle Physics and Department of Physics, Kyushu University, Fukuoka, Japan; 232 LPMR, Faculté des Sciences, Université Mohamed Premier, Oujda, Morocco; 233 Facultad de Ciencias y Centro de Investigaciónes, Universidad Antonio Nariño, Bogotá, Colombia; 234 iThemba Labs, Cape Town, Western Cape South Africa; 235 School of Physics, University of Sydney, Sydney, NSW Australia; 236 Transilvania University of Brasov, Brasov, Romania; 237 Physics Department, National Institute for Research and Development of Isotopic and Molecular Technologies, Cluj-Napoca, Romania; 238 University of West Attica, Athens, Greece; 239 Khalifa University of Science and Technology, Abu Dhabi, United Arab Emirates; 240 Division of Physics, TOBB University of Economics and Technology, Ankara, Türkiye; 241 Graduate School of Science and Technology, Tokyo Metropolitan University, Tokyo, Japan; 242 Present Address: Department of Physics, King’s College London, London, UK; 243 Present Address: Institute of Physics, Azerbaijan Academy of Sciences, Baku, Azerbaijan; 244 Present Address: Imam Mohammad Ibn Saud Islamic University, Riyadh, Saudi Arabia; 245 Present Address: Department of Physics, University of Thessaly, Volos, Greece; 246 Present Address: An-Najah National University, Nablus, Palestine; 247 Present Address: Department of Physics, University of Fribourg, Fribourg, Switzerland; 248 Present Address: Department of Physics, Westmont College, Santa Barbara, CA USA; 249 Present Address: Departament de Fisica de la Universitat Autonoma de Barcelona, Barcelona, Spain; 250 Present Address: University of Siena, Siena, Italy; 251 Present Address: Affiliated with an institute formerly covered by a cooperation agreement with CERN, Geneva, Switzerland; 252 Present Address: The Collaborative Innovation Center of Quantum Matter (CICQM), Beijing, China; 253 Present Address: Faculty of Physics, Sofia University ‘St. Kliment Ohridski’, Sofia, Bulgaria; 254 Present Address: Università di Napoli Parthenope, Naples, Italy; 255 Present Address: Institute of Particle Physics (IPP), Ottawa, ON Canada; 256 Present Address: Department of Physics, Bolu Abant Izzet Baysal University, Bolu, Türkiye; 257 Present Address: Faculty of Physics, University of Bucharest, Bucharest, Romania; 258 Present Address: Borough of Manhattan Community College, City University of New York, New York, NY USA; 259 Present Address: National Institute of Physics, University of the Philippines Diliman (Philippines), Quezon City, Philippines; 260 Present Address: Department of Financial and Management Engineering, University of the Aegean, Chios, Greece; 261 Present Address: TRIUMF, Vancouver, BC Canada; 262 Present Address: Institucio Catalana de Recerca i Estudis Avancats, ICREA, Barcelona, Spain; 263 Present Address: Henan University, Kaifeng, China; 264 Present Address: Physics Department, Yeditepe University, Istanbul, Türkiye; 265 Present Address: Institute of Theoretical Physics, Ilia State University, Tbilisi, Georgia; 266 Present Address: CERN, Geneva, Switzerland; 267 Present Address: Center for Interdisciplinary Research and Innovation (CIRI-AUTH), Thessaloniki, Greece; 268 Present Address: Hellenic Open University, Patras, Greece; 269 Present Address: Department of Modern Physics and State Key Laboratory of Particle Detection and Electronics, University of Science and Technology of China, Hefei, China; 270 Present Address: Department of Mathematical Sciences, University of South Africa, Johannesburg, South Africa; 271 Present Address: Department of Physics, Stellenbosch University, Stellenbosch, South Africa; 272 Present Address: Department of Physics, University of Colorado Boulder, Boulder, CO USA; 273 Present Address: Département de Physique Nucléaire et Corpusculaire, Université de Genève, Geneva, Switzerland; 274 Present Address: Institut für Experimentalphysik, Universität Hamburg, Hamburg, Germany; 275 Present Address: Centre of Physics of the Universities of Minho and Porto (CF-UM-UP), Porto, Portugal; 276 Present Address: Institute for Nuclear Research and Nuclear Energy (INRNE) of the Bulgarian Academy of Sciences, Sofia, Bulgaria; 277 Present Address: Washington College, Chestertown, MD USA; 278 Present Address: Institute of Applied Physics, Mohammed VI Polytechnic University, Ben Guerir, Morocco; 279 Present Address: Department of Physics, Stanford University, Stanford, CA USA; 280 Present Address: Institute of Physics and Technology, Mongolian Academy of Sciences, Ulaanbaatar, Mongolia

**Keywords:** Experimental particle physics, Characterization and analytical techniques

## Abstract

Jet flavour tagging enables the identification of jets originating from heavy-flavour quarks in proton–proton collisions at the Large Hadron Collider, playing a critical role in its physics programmes. This paper presents GN2, a transformer-based flavour tagging algorithm deployed by the ATLAS Collaboration that represents a different methodology compared to previous approaches. Designed to classify jets based on the flavour of their constituent particles, GN2 processes low-level tracking information in an end-to-end architecture and incorporates physics-informed auxiliary training objectives to enhance both interpretability and performance. Its performance is validated in both simulation and collision data. The measured *c*-jet (light-jet) rejection in data is improved by a factor of 3.5 (1.8) for a 70% *b*-jet tagging efficiency, compared to the previous algorithm. GN2 provides substantial benefits for physics analyses involving heavy-flavour jets, such as measurements of Higgs boson pair production and the couplings of bottom and charm quarks to the Higgs boson, and demonstrates the impact of advanced machine learning methods in experimental particle physics.

## Abstract (web summary)

Identifying jets originating from heavy quarks plays a fundamental role in hadronic collider experiments. In this work, the ATLAS Collaboration describes and tests a transformer-based neural network architecture for jet flavour tagging based on low-level input and physics-inspired constraints.

## Introduction

The Large Hadron Collider (LHC)^1^ is the world’s most powerful particle collider. It is used to extend the boundaries of our understanding of fundamental particles and their interactions. It offers a unique opportunity to test the Standard Model (SM) of particle physics, as well as search for new phenomena beyond the Standard Model (BSM). The demanding experimental conditions at the LHC necessitate continuous innovation by the main experiments, pushing them to apply cutting-edge technologies to efficiently identify physics processes of interest within the largest proton–proton (*pp*) collision dataset ever recorded. Hadronic jets, collimated streams of particles initialised by quarks or gluons, are the most abundant physics objects in *pp* collision events, and their characteristics are widely utilised in data analyses.

The flavour of a hadronic jet is determined by the types of hadrons or leptons it contains. Flavour tagging concerns the classification of hadronic jets into those containing *b-*hadrons (*b*-jets), *c-*hadrons (*c-*jets), hadronic *τ-*lepton decays (*τ-*jets), and none of the above (light-jets), using algorithms sensitive to the distinctive properties of the respective classes. Since the beginning of Run 1 of the LHC (2009–2013), the ATLAS experiment^2,3^ has achieved continuous improvement in the performance of these algorithms. The progress has mostly been driven by the integration of machine-learning techniques, including boosted decision trees and neural networks. The state-of-the-art algorithms used thus far to analyse the data at $\sqrt{s}=13\,{{\rm{TeV}}}$ from Run 2 of the LHC (2015–2018)^4,5^ led to very impactful physics results such as the observations of the Higgs boson decaying to bottom quarks^6^ and its production in association with a pair of top quarks^7^. Flavour tagging plays an essential role in the comprehensive research programme of ATLAS, which includes precision measurements of the Higgs boson^8^, top quark^9^ and other SM processes^10^, as well as the searches for supersymmetry^11^ and other BSM phenomena^12^. This work describes a flavour tagging algorithm developed by the ATLAS Collaboration for the analysis of data from *pp* collisions recorded during Run 2 (2015–2018) and Run 3 (2022–2026) of the LHC at centre-of-mass energies of $\sqrt{s}=13\,{{\rm{TeV}}}$ and $\sqrt{s}=13.6\,{{\rm{TeV}}}$, respectively.

Flavour-tagging techniques rely on the long lifetime, high mass, high decay multiplicity and characteristic decay modes of *b-* and *c-*hadrons, and the properties of heavy-quark fragmentation^13^. The typical lifetime of the order of *τ* ≈ 1.5 ps^13–15^ for *b-*hadrons in jets with transverse momenta in the range from tens to hundreds of GeV results in them travelling a mean flight length 〈*l*〉 = *βγcτ* in the range from few millimetres to centimetres before decaying, which often leads to a secondary vertex significantly displaced from the collision point. Displaced vertices can also be produced by *c-*hadrons, which have lifetimes of *τ* ≈ 0.2–1.0 ps, depending on the species^16–18^, and *τ-*leptons, which have a lifetime of *τ* ≈ 0.29 ps but a much lower decay multiplicity^18,19^. The majority of *b*-jets also contain a tertiary vertex from the decay of the *c-*hadron produced in the *b-*hadron decay.

The traditional flavour-tagging algorithms developed by the ATLAS Collaboration are based on a two-stage approach^4,5,20^. In the first step, specialised low-level algorithms employ complementary approaches to extract information from the trajectories of the charged-particle constituents (‘tracks’) associated with the jet. These specialised algorithms either rely on the properties of individual tracks or leverage their correlations with properties of other tracks to explicitly reconstruct displaced vertices. In the second step, the outputs of low-level algorithms are subsequently combined in a high-level multivariate classifier to maximise performance. The most recent algorithm employed by the ATLAS Collaboration, following this paradigm, is a deep neural network (DL1d) that leverages a low-level track-based algorithm (DIPS)^21^ based on Deep Sets^22^. DL1d has already improved the performance by a factor of 1.3 relative to the most advanced algorithm used in published Run-2 physics analyses^5^.

The introduction of graph neural networks for object reconstruction in particle physics experiments^23^ prompted a shift in the design strategy of the ATLAS Collaboration. This led to the development of the General Network (GN) series of flavour-tagging algorithms, which directly process track and jet information and are trained using target labels extracted from Monte Carlo (MC) simulation. In parallel, the CMS Collaboration followed a similar trajectory, evolving from two-stage approaches^24,25^ to unified, end-to-end network architectures^26–28^.

**Fig. 1:**
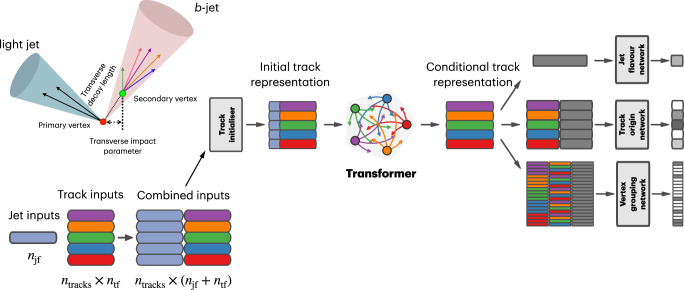
Illustration of the GN2 algorithm with jet and track input variables, discriminating between jet flavours by exploiting secondary vertices and other properties stemming from the displaced decays of *b*-hadrons, in the transverse plane. The jet features are copied for each track associated with the jet. The combined vectors are then fed into a per-track initialisation network, followed by a transformer encoder and a global representation of the jet. *n*_jf_ (*n*_tf_) corresponds to the number of jet (track) features. The pooled jet representation and output track embeddings are provided as inputs to the three task-specific networks. Details of the GN2 architecture are summarised in the ‘Methods’ section.

The ATLAS GN tagger uses jet flavour prediction as its primary training target and introduces auxiliary training objectives to reconstruct the internal structure of a jet by grouping tracks originating from a common vertex and by predicting the underlying physics process from which each track originated. Such physics domain knowledge is embedded in a combined loss function that enables a simultaneous optimisation, instead of relying on manually optimised low-level algorithms. This flexible structure allows the swift re-tuning of the algorithms to suit alternative experimental conditions or physics goals. A demonstrator version, GN1, achieves the above design goals using a graph-neural-network^29^, while the deployment version, GN2, applies a single transformer model^30^, illustrated in Fig. 1. Details of the algorithm architectures are summarised in the ‘Methods’ section, together with descriptions of the ATLAS detector, simulation samples, physics objects, and analysis strategies.

GN2 achieves a remarkable performance boost compared with the DL1d algorithm, with improvements by a factor of 1.5-4 observed in its major experimental applications. The deployment of GN2 should greatly enhance the physics reach of ATLAS in flagship analyses, such as the search for Higgs pair production and the *c*-quark Yukawa coupling measurement, for which the projected sensitivity at the High Luminosity LHC is improved by up to 30%^31^. These improvements do not come with a strong dependence on the choice and configuration of the MC event generator, and are confirmed by measured performance in recorded collisions. The innovative auxiliary training objectives bring excellent interpretability and opens up new avenues for future applications.

To facilitate future developments and strengthen the connections between collider experiments and the broader scientific research community, a subset of the training sample with all the required information to train GN2 can be acquired via the CERN Open Data Portal^32,33^.

## Results

### Algorithm performance in simulation

The performance of a *b*-tagging algorithm is evaluated based on its ability to reject *c*-, *τ*- and light-jets while maintaining a desired *b*-jet tagging efficiency. Similarly, the *c*-tagging performance is assessed by its capability to distinguish *c*-jets from the other jet flavours. The data samples used for training and evaluation of the model must contain jets from all flavour classes. This is achieved using jets sampled from a mixture of simulated top quark pair ($t\overline{t}$) and ${Z}^{{\prime} }$ events, where the latter sample considers a hypothetical heavy BSM particle, ${Z}^{{\prime} }$^34^, which can decay into pairs of *b*-quarks, *c*-quarks, *τ-*leptons or light quarks, to populate jets in the TeV regime. The samples are simulated with MC event generators at centre-of-mass energies of both $\sqrt{s}=13\,{{\rm{TeV}}}$ and $\sqrt{s}=13.6\,{{\rm{TeV}}}$. All simulated events are processed through the ATLAS detector simulation^35^ based on GEANT4^36–38^. Further details on the simulation samples and the jet flavour labelling are discussed in the ‘Methods’ section. A mixture of samples generated at $\sqrt{s}=13.6\,{{\rm{TeV}}}$ and $\sqrt{s}=13\,{{\rm{TeV}}}$ is used in the training, to achieve similar performance in both conditions. In this section, the performance evaluated with Run-3 samples at $\sqrt{s}=13.6\,{{\rm{TeV}}}$ is presented. Jets are classified for *b*-tagging using a single discriminant *D*_*b*_, which combines the algorithm’s jet flavour prediction output probabilities of a jet being a *b*-jet (*p*_*b*_), a *c*-jet (*p*_*c*_), a *τ-*jet (*p*_*τ*_) or a light-jet (*p*_*u*_) and is defined as:

1 $${D}_{b}=\log \left(\frac{{p}_{b}}{{f}_{c}{p}_{c}+{f}_{\tau }{p}_{\tau }+\left(1-{f}_{c}-{f}_{\tau }\right){p}_{u}}\right).$$

A jet is considered *b*-tagged if it has a *D*_*b*_ score larger than a given value. A selection on *D*_*b*_ defines an operating point (OP) associated with a certain inclusive *b*-jet tagging efficiency, calculated as the fraction of *b*-jets that are *b*-tagged. The mis-tagging rate for *c*-, *τ*- and light-jets is determined by the fraction of jets that are mistakenly *b*-tagged, for that given jet flavour, and the rejection is the reciprocal of the mis-tagging rate. The ATLAS Collaboration uses a sample of simulated $t\bar{t}$ events, where most jets have a *p*_T_ below 250 GeV, to derive the OPs. The free parameters *f*_*c*(*τ*)_ determine the relative weighting between *p*_*c*(*τ*)_ and *p*_*u*_ in the discriminant *D*_*b*_. The specific value of *f*_*c*_ is determined through an optimisation procedure aimed at obtaining a certain balance between rejections of *c-*jets and light-jets in simulated $t\overline{t}$ events. The value of *f*_*τ*_ is optimised to maximise the *τ-*jet rejection, while ensuring a negligible impact upon the *c*-jet and light-jet rejection. In the case of GN2, *f*_*c*(*τ*)_ is set to 0.2 (0.05), while for DL1d, which does not have a *τ-*jet output in the model, *f*_*c*_ is set to 0.018. For GN2, *f*_*c*_ is tuned to reach a much higher *c*-jet rejection, while still achieving a better light-jet rejection, compared with DL1d.

**Fig. 2:**
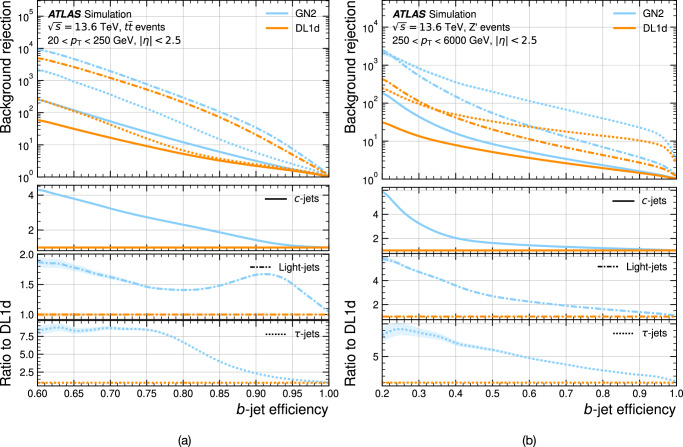
$b$-tagging performance of GN2 and DL1d evaluated in MC simulations. The *c*-jet (solid), light-jet (dotted-dashed), and *τ-*jet (dashed) rejections as a function of the *b*-jet tagging efficiency for **a** jets in the $t\bar{t}$ sample with 20 < *p*_T_ < 250 GeV and **b** jets in the ${Z}^{{\prime} }$ sample with 250 < *p*t < 6000 GeV, for both GN2 (light blue) and DL1d (dark orange). The performance of GN2 with respect to DL1d is shown in the bottom panels. The 68% confidence intervals calculated assuming no correlations between the rejections are indicated by the shaded regions, and the uncertainty on each rejection is obtained according to a binomial distribution.

Figure 2 illustrates the tagger performance in terms of the *c-*jet, light-jet and *τ-*jet rejection as a function of the *b*-jet tagging efficiency. In both the $t\overline{t}$ and ${Z}^{{\prime} }$ samples, GN2 exhibits significantly better background rejection compared with DL1d across the entire range of *b*-jet tagging efficiencies. The degree of improvement depends on the *b*-jet tagging efficiency. In the $t\overline{t}$ sample, the *c*-jet (light-jet) rejection of GN2 improves by more than a factor of 3 (1.6), compared with DL1d, for the most commonly used 70% OP. The performance of both algorithms starts degrading once the jet *p*_T_ reaches around 200 GeV, due to several confounding factors, including suboptimal tracking performance in dense environments where the spatial separation between tracks becomes smaller^39^. In the ${Z}^{{\prime} }$ sample, applying the 70% OP selection on *D*_*b*_ yields a *b*-jet tagging efficiency of 30%, and the *c*-jet (light-jet) rejection of GN2 improves by more than a factor of 3 (4), compared with DL1d. The inclusion of a *τ-*jet output node in GN2 leads to an even greater enhancement in the *τ-*jet rejection, by up to a factor of 8 (9) for jets in the $t\overline{t}$ (${Z}^{{\prime} }$) sample, without significantly degrading the *c*-jet and light-jet rejection.

The performance of a *c*-tagging algorithm is evaluated based on its ability to reject *b*-, *τ*- and light-jets while maintaining a desired *c*-jet tagging efficiency. Due to the end-to-end architecture that does not rely on low-level tagger inputs, GN2 can seamlessly be adapted as a *c*-tagging algorithm without re-training any lower level algorithms. Similar to *b-*tagging, a discriminant, *D*_*c*_, is constructed as:

2 $${D}_{c}=\log \left(\frac{{p}_{c}}{{f}_{b}{p}_{b}+{f}_{\tau }{p}_{\tau }+\left(1-{f}_{b}-{f}_{\tau }\right){p}_{u}}\right),$$

where *f*_*b*(*τ*)_ is the free parameter that controls the flavour composition of the background in the background hypothesis. The value chosen for *f*_*b*(*τ*)_ is 0.3 (0.01) for GN2, while for DL1d, *f*_*b*_ is 0.1, following a similar optimisation procedure as for *D*_*b*_.

**Fig. 3:**
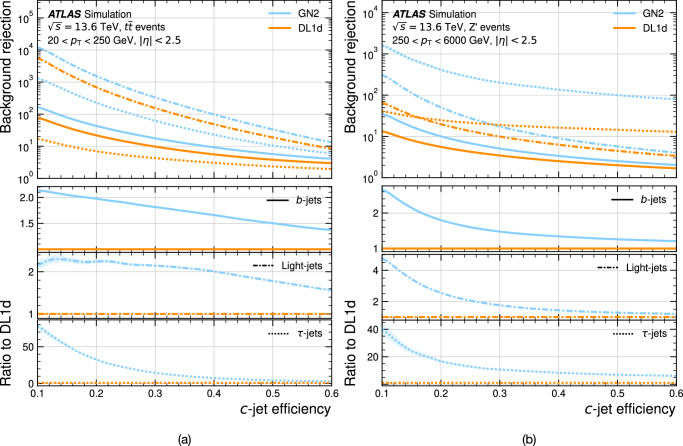
$c$-tagging performance of GN2 and DL1d evaluated in MC simulations. The *b*-jet (solid), light-jet (dotted-dashed), and *τ-*jet (dashed) rejections as a function of the *c*-jet tagging efficiency for **a** jets in the $t\bar{t}$ sample with 20 < *p*_T_ < 250 GeV and **b** jets in the ${Z}^{{\prime} }$ sample with 250 < *p*t < 6000 GeV, for both GN2 (light blue) and DL1d (dark orange). The performance of GN2 relative to DL1d is shown in the bottom panels. The 68% confidence intervals calculated assuming no correlations between the rejections are indicated by the shaded regions, and the uncertainty on each rejection is obtained according to a binomial distribution.

The *c-*tagging performance of DL1d and GN2 are compared in Fig. 3, which shows a significant improvement in performance across all *c*-jet tagging efficiencies. The *b*-jet (light-jet) rejection is enhanced by a factor of approximately 1.8 (2.2) in the $t\overline{t}$ sample at a 30% *c*-jet tagging efficiency, which is a typical choice in measurements of the *c*-quark Yukawa coupling^40^. The *b*-jet (light-jet) rejection is increased by a factor of approximately 2.7 (4.7) in the ${Z}^{{\prime} }$ sample at a corresponding efficiency of 10%. The *τ*-jet rejection is improved by a factor of approximately 15 (40) in the $t\overline{t}$ (${Z}^{{\prime} }$) sample.

### Algorithm performance in collision data

Due to imperfections in the physics modelling of the MC generator and in the simulated detector response, the distribution of the input variables to the algorithms and their correlations differ between collision data and simulation, resulting in a performance difference. It is not practical to correct each individual mis-modelled variable, so dedicated calibration analyses are employed to measure the tagging efficiency of *b*-jets, *c-*jets and light-jets for pre-defined OPs directly^4,41,42^. In the case of the GN2 algorithm, five OPs are defined corresponding to inclusive *b*-jet tagging efficiencies of 65%, 70%, 77%, 85% and 90% while for DL1d four OPs are constructed corresponding to inclusive *b*-jet tagging efficiencies of 60%, 70%, 77% and 85%. The results presented in this paper are derived using *pp* collision data recorded during Run 2 of the LHC at $\sqrt{s}$ = 13 TeV, corresponding to an integrated luminosity of 140 fb^−1^. The tagging performance in data for *b*-jets, *c-*jets, and light-jets is measured, in order to obtain jet-flavour-dependent simulation-to-data correction factors, binned in jet *p*_T_. They are applied to MC-simulated jets to rescale their tagging efficiencies and mis-tagging rates to match those measured in collision data. The calibration of *b*-jets and *c-*jets is done with $t\bar{t}$ events^4,41^, while the calibration of light-jets is performed using jets produced in association with a *Z* boson^42^. Details of the calibration analyses are provided in the ‘Methods’ section.

**Fig. 4:**
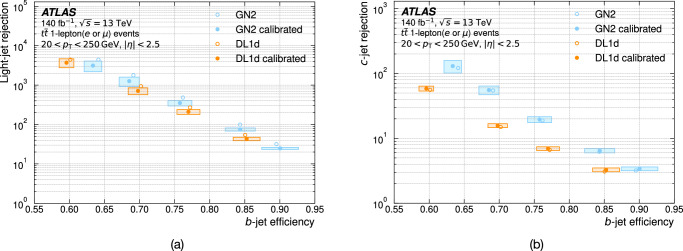
$b$-tagging performance of GN2 and DL1d measured in data and MC simulations. The **a** light-jet rejection and **b** *c*-jet rejection as a function of the *b*-jet tagging efficiency for GN2 (light blue) and DL1d (dark orange), directly obtained in simulation (hollowed circle) and rescaled to match those in collision data (solid point). The horizontal error bands correspond to the uncertainties associated with the *b*-jet tagging efficiency measurement, while the vertical error bands indicate the uncertainties associated with the rejection measurements. A $t\overline{t}$ MC simulation sample with a reconstructed electron or muon is used to derive these results.

Figure 4 presents the calibrated tagging efficiencies and rejections of GN2 and DL1d, along with their associated uncertainties, for each OP. The inclusive efficiencies and rejections are obtained by averaging over the events in a simulated $t\overline{t}$ sample after requiring the presence of one reconstructed electron or muon. The original efficiencies from the simulated sample are included as references, enabling a direct comparison that shows similar agreement between data and simulation for both GN2 and DL1d. The GN2 tagger demonstrates clear improvements over DL1d in collision data. For instance, the measured *c*-jet (light-jet) rejection in data is increased by a factor of 3.5 (1.8) for the 70% OP. The measurements in data provide conclusive evidence of the enhanced performance enabled by advanced machine-learning algorithms in identifying heavy-flavour jets at the LHC.

## Discussion

Key challenges with machine-learning algorithms based on low-level inputs, such as GN2, include the potential loss of interpretability and the need to ensure consistent performance across different MC simulation methods. Robustness against these potential shortcomings is critical to prevent the algorithm from relying on unphysical features of the training sample. In this section, these aspects are discussed further.

### Physics inspiration and the auxiliary training objectives

A key strength of the GN2 model lies in its physics-inspired constraints, which aid the main task of jet classification while also improving the interpretability of the model. This is accomplished by incorporating two additional training objectives: predicting the origin of tracks associated with the jet and determining which tracks originate from common vertices. These objectives are not strictly necessary for the jet classification task and are therefore referred to as auxiliary training objectives. The technical implementation details are provided in the ‘Methods’ section.

The track classification auxiliary training objective aims to estimate the probability that a track originates from one of the following physical processes: a pile-up interaction^43^; the primary hard-scatter interaction; the decay of a *b-*hadron; the decay of a *c-*hadron produced by a *b-*hadron; the decay of a *c-*hadron; the decay of a *τ*-lepton; or any other secondary source. Class-weighted losses are applied during training to mitigate the class imbalance, and tracks are classified by the highest-probability category during evaluation. The class weights are fixed and based on the inverse class frequencies in the training dataset.

The classification efficiency refers to the probability for the track’s origin to be correctly predicted, in a group of tracks with certain target origins, while the purity corresponds to the fraction of correctly predicted tracks, within a group of tracks with specific predicted origins. When combining the two categories involving a *b-*hadron, GN2 achieves an efficiency (purity) of 84% (84%). For tracks that are not of heavy-flavour (HF) origin, the efficiency (purity) is 85% (96%). The above performance is evaluated in Run-3 samples at $\sqrt{s}=13.6\,{{\rm{TeV}}}$.

The vertex finding auxiliary training objective aims to identify groups of tracks that originate from a common spatial point. Each pair of tracks in the jet is classified to determine whether they share the same vertex. Using these pair-wise compatibility scores, track groups (vertices) are formed via a union-find algorithm^44^. SV1, an existing secondary vertex reconstruction algorithm detailed in ref. ^5^, serves as a reference algorithm. SV1 reconstructs a single inclusive vertex, whereas GN2 can identify multiple vertices of various types within a jet. Therefore, an aggregation procedure is applied to the output of GN2 to enable a direct comparison with the single inclusive vertex produced by SV1. To study the vertex properties of *b*-jets, the identified vertex containing the most tracks that have a predicted primary origin is removed, as this is likely to be the vertex associated with the primary hard-scatter interaction. Next, the remaining GN2 vertices that include at least one track predicted to have a HF origin are consolidated into a single inclusive vertex.

**Fig. 5:**
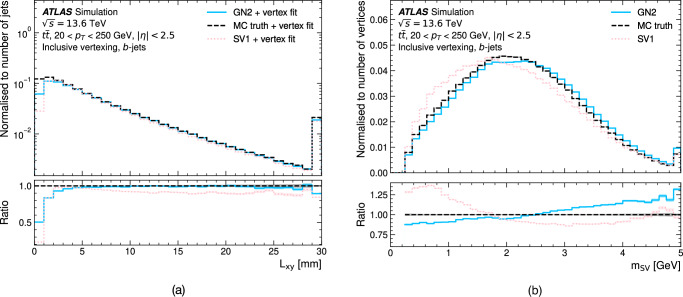
Secondary vertex properties reconstructed using tracks grouped by the GN2 and SV1 algorithms. The **a** transverse displacement and the **b** mass of the secondary vertex obtained by the GN2 (solid) and the SV1 (dotted) algorithms. While the transverse displacement is calculated via a Billoir fit performed on the tracks assigned to the vertex by the respective algorithm, the vertex mass is defined as the invariant mass of the same set of assigned tracks. MC truth (dashed) corresponds to an inclusive reference vertex derived from all tracks associated to simulation-level vertices containing only *b-*hadron tracks. The last bin in each plot includes overflow.

An inclusive reference vertex is constructed in simulated events, by combining all tracks from simulation-level secondary vertices within the jet that consist solely of HF tracks. A Billoir fit^45^ is performed on the tracks selected by the GN2 and SV1 vertex finding algorithms to obtain the transverse displacement of the vertex, *L*_*xy*_. Figure 5 presents the *L*_*xy*_ distribution for vertices obtained with GN2 and DL1d in *b*-jets from a simulated $t\overline{t}$ sample, compared to the expected distribution derived from the inclusive reference vertex. GN2 consistently achieves higher vertex-finding efficiency than SV1 across the entire distribution of *L*_*xy*_. The mass of the secondary vertex can also be calculated using the momenta of tracks selected by the vertex finding algorithms. The distribution of the secondary vertex mass normalised to unity is also shown in Fig. 5. Remarkably, the mass of the secondary vertices reconstructed by GN2 exhibits good agreement with the mass of the inclusive reference vertex, despite the vertex mass not being explicitly targeted during training. Unlike SV1, GN2 does not impose explicit selections on track properties such as impact parameters. This leads to a higher efficiency, albeit with a small contamination from non-HF tracks, which results in a slightly larger secondary vertex mass.

**Fig. 6:**
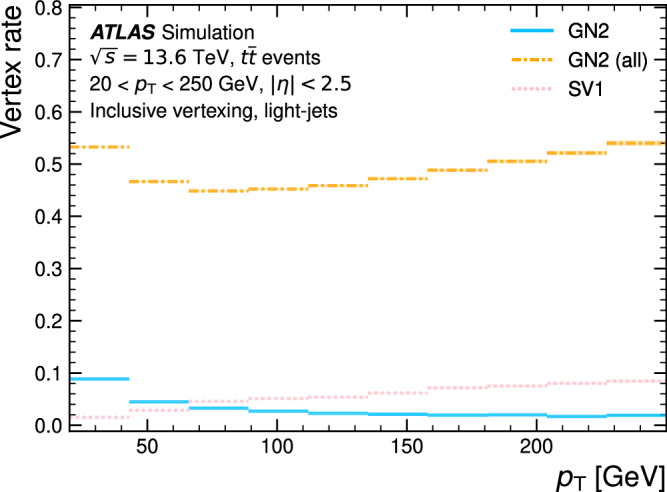
The rate of inclusive vertices reconstructed by the GN2 algorithm in light-jets as a function of the jet *p*_T_, without any selections (dotted-dashed) and with the requirement of the vertex containing at least one track with predicted HF origin (solid). Results from the SV1 algorithm are added as a reference (dotted). The 68% confidence intervals calculated according to a binomial distribution are indicated by the shaded regions.

GN2 identifies all types of vertices including those from material interactions, photon conversions, and in-flight decays of light hadrons. Consequently, the rate of vertices reconstructed in light-jets, defined as the fraction of light-jets containing a GN2 inclusive vertex, is expected to be much higher compared to SV1 if no selections are applied in the aggregation procedure described above. Figure 6 confirms this with light-jets in the simulated $t\overline{t}$ sample and shows that once requiring the GN2 inclusive vertex to contain at least one track with predicted HF origin, the vertexing rate is dramatically reduced, down to the same level as SV1.

To test the impact of the auxiliary objectives on the performance of the main jet classification task, various GN2 configurations are trained and tested. The resulting *c*-jet and light-jet rejections are reduced by up to 30% in both the $t\overline{t}$ and ${Z}^{{\prime} }$ samples, if both auxiliary objectives are disabled. Disabling only one of them is sufficient to recover most of the performance loss, indicating that the two tasks are highly correlated in their contributions to the main jet flavour tagging objective.

Although the outputs from the auxiliary tasks described above mainly serve as a way to improve HF jet identification, with future development, their direct usage in physics analyses remains a promising possibility.

### Robustness against generator modelling variations

Flavour-tagging algorithms are sensitive to the modelling of parton showering, hadronisation, the underlying event and the properties of heavy-hadron decays^46^. To evaluate the robustness of the algorithm against modelling variations, a comparative study of the GN2 performance in the nominal simulated $t\overline{t}$ sample used during training and samples produced with alternative generator settings, both with Run-2 conditions at $\sqrt{s}=13\,{{\rm{TeV}}}$, is performed.

The event and showering generators adopted for the nominal sample are Powhegbox^47–50^ and Pythia^51^, respectively. The alternative samples include the use of a different showering generator (Herwig^52–54^), whilst keeping the same event generator, and the use of Sherpa^55^, which applies a different approach to all parts of the event generation model. The ratio between the efficiency obtained with an alternative generator setup and with the nominal setup is used to quantify the generator dependence of the algorithms.

Table 1 shows these ratios for *b*-jets, *c-*jets, and light-jets at the 70% and 85% OPs. Across the tested generators, the GN2 performance for *b*-jets agrees to within 1–2%, for *c-*jets the agreement is within 10%, and for light-jets the agreement is within 4%. Similar agreement is also observed for other OPs. The level of relative disagreement between DL1d and GN2 is close to unity, suggesting that despite the GN2 model being significantly more complex, it does not induce additional generator dependence.

**Table 1:** Ratios of the efficiencies obtained with samples using alternative MC generators, relative to those in the nominal Powhegbox + Pythia sample used during training of the algorithm

		70% OP		85% OP	
		DL1d	GN2	DL1d	GN2
Powhegbox + Herwig	*b*-jets	0.984	0.984	0.989	0.990
	*c-*jets	0.951	0.904	0.977	0.983
	light-jets	1.003	1.000	1.015	1.011
Sherpa	*b*-jets	0.996	0.995	0.992	0.992
	*c* -jets	0.923	0.938	0.947	0.931
	light-jets	1.039	1.013	1.077	1.042

Statistical uncertainties from evaluating the same algorithm on different samples are negligible and thus not shown.

## Methods

### The ATLAS detector

The ATLAS experiment^2,3^ at the LHC is a multipurpose particle detector with a forward-backward symmetric cylindrical geometry and a solid-angle coverage of almost 4*π*. It is used to record particles produced in *pp* collisions at the LHC through a combination of particle position and energy measurements. It includes an inner-tracking detector (ID) surrounded by a thin superconducting solenoid providing a 2 T axial magnetic field, electromagnetic and hadronic calorimeters, and a muon spectrometer. The ID consists of silicon pixel, silicon microstrip, and transition radiation tracking detectors. The muon spectrometer surrounds the calorimeters and is based on three large superconducting air-core toroidal magnets with eight coils each providing a field integral of between 2 T m and 6 T m across the detector.

An extensive software suite^56^ is used in data simulation, the reconstruction and analysis of real and simulated data, detector operations, and the trigger and data acquisition systems of the experiment.

### Monte Carlo simulation samples

The $t\overline{t}$ events at $\sqrt{s}=13\,{{\rm{TeV}}}$ are modelled using the Powhegbox[v2]^47–50^ event generator at next-to-leading-order (NLO) in the strong coupling constant *α*_s_ with the NNPDF3.0 NLO^57^ parton distribution function (PDF) set and the first-gluon-emission cut-off scale parameter *h*_damp_ set to 1.5*m*_*t*_, with a top-quark mass of *m*_*t*_ = 172.5 GeV. Parton shower, hadronisation, and the underlying event are modelled by interfacing Powhegbox[v2] to Pythia 8.230^51^, using the A14 set of tuned parameters^58^ and the NNPDF2.3LO PDF set^59^. The decays of *b*- and *c*-hadrons are performed by Evtgen 1.6.0^60^.

The ${Z}^{{\prime} }$ events at $\sqrt{s}=13\,{{\rm{TeV}}}$ used to enrich the dataset with high-*p*_T_ jets are generated using Pythia 8.243 with the A14 set of tuned parameters for the underlying event and the leading-order (LO) Nnpdf 2.3LO PDF set. A broad jet *p*_T_ spectrum with an almost uniform distribution between 250 GeV and 1.5 TeV and a tail expanding to 6 TeV is obtained by applying a weighting factor that modifies the original cross-section of the ${Z}^{{\prime} }$ resonance. The decays to $b\bar{b}$, $c\bar{c}$, and light-flavour quark pairs are set to have equal branching ratios, while the branching ratio to $\tau \bar{\tau }$ is set to 5%. The decays of *b*- and *c*-hadrons are performed by Evtgen 1.7.0.

The $t\overline{t}$ and ${Z}^{{\prime} }$ events at $\sqrt{s}=13.6\,{{\rm{TeV}}}$ are produced using the same setups, but with newer versions of Pythia (8.308) and Evtgen (2.1.1).

The impact of using different generators and models for parton shower and hadronisation is studied with simulated $t\overline{t}$ events from alternative generator setups. Two scenarios are considered, where either only the showering algorithm is varied, or the entire chain is changed. The former is achieved by interfacing the Powhegbox[v2] generator with the Herwig[7.2.1]^52–54^ showering algorithm using the Herwig[7.1] default set of tuned parameters, with the Nnpdf 3.0 NLO set of PDFs. The latter is realised with the Sherpa[2.2.12]^55^ generator, using NLO-accurate matrix elements for up to one additional parton, and LO-accurate matrix elements for up to four additional partons, calculated with the COMIX^61^ and Openloops^62–64^ libraries. The Sherpa parton shower^65,66^ is applied using the Meps@nlo prescription^67–70^ and the set of tuned parameters developed by the Sherpa authors to match the Nnpdf 3.0 NNLO set of PDFs.

### Objects for flavour tagging

ATLAS uses a right-handed coordinate system with its origin at the nominal interaction point in the centre of the detector and the *z*-axis along the beam pipe. The *x*-axis points from the nominal interaction point to the centre of the LHC ring, and the *y*-axis points upwards. Cylindrical coordinates (*r*, *ϕ*) are used in the transverse plane, *ϕ* being the azimuthal angle around the *z*-axis. The pseudorapidity is defined in terms of the polar angle *θ* as $\eta=-\ln \tan (\theta /2)$. Angular distance is measured in units of $\Delta R\equiv \sqrt{{(\Delta \eta)}^{2}+{(\Delta \phi)}^{2}}$.

The fundamental objects for flavour tagging are jets, tracks, and vertices. A concise description of these objects is provided below, while a detailed description is available in ref. ^5^.

Tracks are reconstructed from ID information^39,71^. To be considered for jet flavour tagging they are required to be within ∣*η*∣ < 2.5, have *p*_T_ > 0.5 GeV and satisfy criteria designed to reject fake and poorly measured tracks^72^.

Primary vertices (PVs) are reconstructed from tracks in the luminous region of the colliding LHC beams using an adaptive multi-vertex filter^73,74^. The PV with the highest sum of squared transverse momenta *p*_T_ of contributing tracks is selected as the primary interaction point (IP) and provides the reference point in an event. The distance of closest approach of a track to the IP, the ‘perigee’, is indicated in the transverse plane by the transverse impact parameter *d*_0_. The longitudinal separation between the IP and the point on the track where *d*_0_ is measured, is indicated by the longitudinal impact parameter *z*_0_. Tracks with large impact parameters can indicate the presence of displaced decays, providing vital information to the flavour tagging algorithms.

The DIPS and GN2 algorithms require tracks to be reconstructed from at least 8 hits in the silicon detector, at most one of which contributes to two tracks, at most two ‘holes’ in the silicon detector, and at most one hole in the pixel detector, where hole denotes a hit missing where one is expected from the track trajectory. Further, requirements on the track impact parameters, ∣*d*_0_∣ < 3.5 mm and $| {z}_{0}\sin \theta | < 5\,{{\rm{mm}}}$, retain charged particle tracks originating from HF hadron decays while suppressing tracks from other sources.

Jets are reconstructed using the anti-*k*_*t*_ algorithm^75^ with radius parameter *R* = 0.4 using the ‘fastjet’ package^76^. The input constituents are ‘particle-flow’ objects^77^ which combine signals in the ATLAS calorimeters and ID to exploit precision tracking information for low-*p*_T_ charged hadrons spatially matched with calorimeter energy deposits. The jet *p*_T_ is corrected to the corresponding particle-level jet *p*_T_ using calibration techniques described in ref. ^78^. The jets are required to have *p*_T_ > 20 GeV (to be within the valid calibration range) and ∣*η*∣ < 2.5 (to be within the tracking fiducial volume set by the ID acceptance) to be considered for flavour tagging. Additionally, jets from pile-up interactions are suppressed by the ‘jet vertex tagger’ (JVT) algorithm^79^, which uses the ID tracks associated with the jet to form a multivariate discriminant. The JVT efficiency for jets originating from the IP is 92% in the simulation. The jet axis, derived from the sum of the momenta of the jet constituents, is used when associating tracks with the jet and when assigning a lifetime sign to the tracks’ impact parameters. Tracks are associated with a given jet by setting a maximum allowed angular separation Δ*R* between the track momenta, defined at the perigee, and the jet axis. The Δ*R* requirement varies as a function of the jet *p*_T_ to account for decay products from *b*-hadrons with larger *p*_T_ being more collimated, ranging from 0.45 for jet *p*_T_ = 20 GeV to 0.26 for jet *p*_T_ > 150 GeV. If a track can be associated with multiple jets, it is assigned to the jet closest in Δ*R*. The sign convention for the lifetime-signed impact parameters assigns a positive sign if the track intersects the jet axis in the transverse plane in front of the IP, and a negative sign if the intersection lies behind the IP^20^. The flavour labels of jets in simulation are assigned depending on the hadrons associated with the jet. The set of weakly decaying hadrons and hadronically decaying *τ-*leptons with *p*_T_ > 5 GeV within a Δ*R* < 0.3 cone around the jet axis determines the jet flavour following a sequential labelling decision tree. A jet is labelled a *b*-jet if it contains at least one *b-*hadron with *p*_T_ > 5 GeV, a *c*-jet (*τ-*jet) if it contains at least one *c-*hadron (hadronic *τ-*lepton decay) and no *b-*hadron, and otherwise it is called a light-jet, where the latter is an inclusive label for the jets originating from a light quark or gluon. These labels are used both for training the algorithms, and for evaluating their performance.

Targets for the auxiliary training objectives are obtained from the simulation-level event record. Tracks are matched with simulation-level particles using the approach in ref. ^39^. Track-origin labels are obtained by analysing the decay history of the matched particles, while track-pair-compatibility labels are obtained by considering the production vertices of the matched particles. Production vertices within 0.1 mm in 3D space are merged to account for the finite resolution of the detector, and the matched track-pairs are assigned the same label.

### The algorithm architecture

The primary flavour tagging algorithm presented is GN2, which directly learns from the charged particle tracks via a transformer-based model. Another algorithm, DL1d, which follows previous approaches of combining inputs from several low-level taggers in a multivariate technique, is also discussed as a baseline reference.

Both algorithms are trained on a dataset created from combining the simulated $t\overline{t}$ and ${Z}^{{\prime} }$ samples described earlier in this section. Jets with 20 GeV < *p*_T_ < 250 GeV are taken from the $t\overline{t}$ sample and those with 250 GeV < *p*_T_ < 6 TeV from the ${Z}^{{\prime} }$ sample. The *b*-jets, light-jets and *τ-*jets are re-sampled in *p*_T_ and *η* to match the corresponding *c*-jet distributions, thereby preventing the models from discriminating between jet flavours based on relative kinematic differences. All input variables to the algorithm training are normalised to have zero mean and unit variance. A coarse optimisation of hyperparameters, such as the number of layers, is carried out for both algorithms, and the AdamW^80^ (Adam^81^) optimiser is used for training GN2 (DL1d) with the learning rate and optimisation schedule defined below.

The GN2 algorithm is an end-to-end architecture without any intermediate taggers involved, as illustrated in Fig. 1. It is based on the GN1^29^ demonstrator version of the algorithm, replacing the Graph Attention Network^82^ with a Transformer^30^ along with other architecture optimisations. GN2 directly accepts information about the jet and associated tracks that are provided by the standard event reconstruction. This results in a simpler and more flexible algorithm which can be easily reoptimised for different physics objectives, such as the identification of highly energetic Higgs bosons decaying into *b*- or *c*-quark pairs^83^, jet energy regression^84^, exotic jet tagging^85^, and jet flavour tagging in the ATLAS high-level trigger^86^. Additionally, when compared with DL1d, GN2 is trained to recognise an additional class of jets that originate from hadronic *τ-*lepton decays.

First, the jet features are concatenated with a fixed-size array of 40 track feature vectors, with unused elements masked when fewer than 40 tracks are available, allowing it to handle variable track multiplicity without zero-padding. Tracks with smaller absolute track impact parameter significance^5^ are dropped if there are more than 40 tracks. The same inputs as for GN1^29^ are used, except the variables related to holes in the silicon tracker, which were found to have no impact on performance. A complementary interpretability analysis using integrated gradients shows that the impact parameter significances and angular variables emerge as particularly influential^87^. The combined vectors are then fed into a per-track initialisation network, which is composed of a single hidden layer and an output layer of size 256. Next, a four layer transformer encoder with eight attention heads is used to produce track representations that incorporate information from other tracks inside the jet. The transformer has an embedding size of 256 and a feed-forward dimension of 512, and uses pre-LayerNorm^88^. After the transformer encoder, the output track representations are projected down to dimension 128, and a global representation of the jet is produced using attention pooling^89^. The pooled jet representation and output track embeddings are provided as inputs to the three task-specific networks. The primary objective, jet classification, uses only the pooled jet representation and has an output layer of size 4, providing *p*_*b*_, *p*_*c*_, *p*_*u*_ and *p*_*τ*_ for the final discriminant definition. The two auxiliary objectives introduced in the Discussion section take advantage of the track embeddings, in addition to the global jet representation. The track origin classification task uses individual track embeddings and has 7 output categories, while the track-pair compatibility task employs a binary output layer, using the embeddings of each pair of tracks. Each task-specific network consists of three hidden layers with size 128, 64 and 32, respectively. ReLU activation^90^ is used throughout the model. Cross-entropy loss is used by all three task-specific networks, which is combined with tunable weights to form the final loss function, enabling a simultaneous optimisation of the entire algorithm. GN2 applies the same auxiliary network structures and loss weights as GN1^29^.

GN2 is trained using a 4-fold strategy to prevent memorisation of the training samples, given their possible use in ATLAS physics analyses. Jets are assigned to one of the four folds pseudo-randomly, with a number seeded by the event number and discrete jet properties. Four classifiers are then trained, each excluding one of the four folds from the training dataset. In physics analysis, each jet is tagged using the classifier it was excluded from during training. Each of the four networks has approximately 2.3M trainable parameters and is trained using approximately 45M (18M) *b*-jets, 45M (18M) *c*-jets, 90M (36M) light-jets and 6.25M (2.5M) *τ*-jets from the $t\overline{t}$ (${Z}^{{\prime} }$) sample, simulated at both $\sqrt{s}=13\,{{\rm{TeV}}}$ and $\sqrt{s}=13.6\,{{\rm{TeV}}}$, with a mixing ratio of 2:1. A learning rate scheduler with cosine annealing^91^ is used with the initial learning rate set to 1 × 10^−7^, which is increased to 5 × 10^−4^ after the first 1% of training steps have been completed. It reduces to 1 × 10^−5^ over the remainder of the training run. A weight decay of 1 × 10^−5^ is also added. A batch size of 12,000 is adopted. The different folds have compatible performance within statistical uncertainty. The training data is translated from a standard ATLAS format^56^ to HDF5^92^. The network is trained with PYTORCH LIGHTNING^93–95^, consuming roughly 300 GPU hours on an NVIDIA A100 card. It is deployed in ATLAS software with ONNXRUNTIME^96^, adding negligible CPU time. With the updated architecture and training setup, the *c*-jet (light-jet) rejection is improved by a factor of 1.5 (1.7) for a 70% *b*-jet tagging efficiency, in the $t\overline{t}$ sample, and by a factor of 1.3 (1.4) for the corresponding 30% *b*-jet tagging efficiency, in the ${Z}^{{\prime} }$ sample.

The DL1d algorithm inherits the architecture from its predecessor DL1r, described in Ref. ^5^, but processes track impact parameters with the DIPS algorithm based on DeepSets^21,22^ instead of a recurrent neural network^97^. Overall, 44 input features are fed into DL1d, including the jet *p*_T_ and *η*. The architecture of DL1d includes eight hidden layers of size 256, 128, 60, 48, 36, 24, 12, and 6, each followed with ReLU activation and batch normalisation. The training was performed with a learning rate of 1 × 10^−3^ and training batch size of 15,000. The training data pipeline is similar to GN2, with the exception that training is done with KERAS and TENSORFLOW^98^ via UMAMI, a dedicated Python toolkit^99^, and deployed in the ATLAS software with LWTNN^100^.

### Performance measurement strategies in collision data

The measurement of the *b*-jet tagging efficiency in collision data is carried out in a roughly 90% pure sample of $t\bar{t}$ events where both top quarks decay leptonically into a lepton, a neutrino and a *b*-quark. The events are required to contain exactly one electron and one muon of opposite charge, in addition to two jets. The invariant masses of the two lepton-jet pairs are used to define one region enriched in *b*-jets and three control regions (CRs). The *b*-jet-enriched region is determined by requiring that both lepton-jet pairs have invariant masses compatible with an on-shell top quark decay. The CRs are used in a likelihood fit to constrain the predicted jet flavour composition. They are constructed to have increased fractions of non-*b*-jets by requiring that at least one or both of the lepton-jet pairs do not originate from the same top-quark decay. The analysis employs a statistical model based on a likelihood function that extracts the efficiency in collision data binned in *p*_T_ for all the *b*-jets in the sample. The dominant systematic uncertainty comes from the modelling of $t\bar{t}$ events. Additional details on the *b*-jet calibration procedure are available in ref. ^4^.

The calibration measurement of the *c*-jet mis-tagging rate is performed in $t\bar{t}$ events where one top quark decays leptonically while the other top quark decays hadronically. A sample of *c-*jets is obtained through the ${W}^{\pm }\to c\bar{s}(\bar{c}s)$ decay from the hadronically decaying top quark. A likelihood-based kinematic reconstruction is employed to find, among the four jets in the event, two jets associated with the hadronically decaying *W*-boson and two jets stemming from the *b*-quarks produced in the top quark decays. The mis-tagging rate of *c*-jets is determined by minimising a *χ*^2^ function computed in bins of the jet *p*_T_ of the two jets from the *W*-boson decay. Additional terms that correct for the potential mis-modelling of the total number of events in each jet *p*_T_ bin are estimated simultaneously from the fit to collision data, while the contribution of background events, in which no *c-*jets are associated with the *W*-boson decay, is estimated from simulations. The mis-tagging rate of light-jets in this sample is corrected using the method described below. As with the *b*-jets calibration analysis, the leading source of systematic uncertainties is the modelling of $t\bar{t}$ events. The *c*-jet mis-tagging rate calibration procedure is detailed in ref. ^41^.

The mis-tagging rate for light-jets is determined using jets produced in association with a *Z* boson, where the *Z* boson decays into muon or electron pairs. The key challenge in this calibration is to develop a method capable of extracting a light-jet mis-tagging rate in data despite the high rejection of the taggers. The method used in this work involves exploiting transformed track variables in alternate taggers that provide reduced *b*(*c*)-jet tagging efficiency and almost unchanged light-jet rejection. The mis-tagging rate of this modified tagger is measured from a fit to the flavour-sensitive secondary vertex mass distribution in collision data, and dedicated uncertainties are introduced so that it can be extrapolated to that of the nominal tagger. These extrapolation uncertainties are a leading source of systematic uncertainty. A detailed description of the procedure is provided in ref. ^42^.

## Supplementary information

Transparent Peer Review file

## Data Availability

Raw data were generated by the ATLAS experiment. Derived data supporting the findings of this study are available from the ATLAS Collaboration upon request. A subset of the training sample and instructions to train GN2 can be acquired via the CERN Open Data Portal^32,33^.

## Code availability

The ATLAS data reduction software is available at *Zenodo* (10.5281/zenodo.4772550)^102^. The GN2 training software suite can be found at *JOSS* (https://joss.theoj.org/papers/10.21105/joss.07217)^95^, and the DL1d training software stack is available at *JOSS* (https://joss.theoj.org/papers/10.21105/joss.05833)^99^.
